# Asymmetric neuromodulation in the respiratory network contributes to rhythm and pattern generation

**DOI:** 10.3389/fncir.2025.1532401

**Published:** 2025-07-08

**Authors:** Rishi R. Dhingra, Peter M. MacFarlane, Peter J. Thomas, Julian F. R. Paton, Mathias Dutschmann

**Affiliations:** ^1^Division of Pulmonary, Critical Care and Sleep, Department of Medicine, Case Western Reserve University, Cleveland, OH, United States; ^2^The Florey Department of Neuroscience and Mental Health, University of Melbourne, Parkville, VIC, Australia; ^3^Department of Pediatrics, Rainbow Babies and Children’s Hospital, Case Western Reserve University, Cleveland, OH, United States; ^4^Department of Mathematics, Applied Mathematics and Statistics, Case Western Reserve University, Cleveland, OH, United States; ^5^Department of Physiology, Manaaki Manawa – The Centre for Heart Research, University of Auckland, Auckland, New Zealand

**Keywords:** breathing, opioid-induced respiratory depression, multi-electrode array, Hopfield network, rhythm generation, central pattern generator

## Abstract

Like other brain circuits, the brainstem respiratory network is continually modulated by neurotransmitters that activate slow metabotropic receptors. In many cases, activation of these receptors only subtly modulates the respiratory motor pattern. However, activation of some receptor types evokes the arrest of the respiratory motor pattern as can occur following the activation of μ-opioid receptors. We propose that the varied effects of neuromodulation on the respiratory motor pattern depend on the pattern of neuromodulator receptor expression and their influence on the excitability of their post-synaptic targets. Because a comprehensive characterization of these cellular properties across the respiratory network remains challenging, we test our hypothesis by combining computational modeling with ensemble electrophysiologic recording in the pre-Bötzinger complex (pre-BötC) using high-density multi-electrode arrays (MEA). Our computational model encapsulates the hypothesis that neuromodulatory transmission is organized asymmetrically across the respiratory network to promote rhythm and pattern generation. To test this hypothesis, we increased the strength of subsets of neuromodulatory connections in the model and used selective agonists *in situ* while monitoring pre-BötC ensemble activities. The *in silico* simulations of increasing slow inhibition were consistent with experiments examining the effect of systemic administration of the 5HT1aR agonist 8-OH-DPAT. Similarly, the effects of increasing slow excitation in the model were experimentally confirmed in pre-BötC ensemble activities before and after systemic administration of the *μ*-opioid receptor agonist fentanyl. We conclude that asymmetric neuromodulation can contribute to respiratory rhythm and pattern generation and accounts for its varied effects on breathing.

## Introduction

Neuromodulation is essential for adaptive function in brain circuits (Dayan, 2012; Marder, 2012; Nadim and Bucher, 2014; Nusbaum et al., 2017). Neuromodulatory transmitters act via metabotropic receptors coupled to intracellular signaling cascades to slowly modify synaptic and membrane properties thereby altering circuit computations mediated by fast synaptic neurotransmission (Brezina, 2010). For example, phasically active midbrain dopaminergic neurons encode a reward prediction error signal that modulates excitability, and hence, activity-dependent plasticity in their target populations (Dayan, 2012).

Given the coordination of breathing with other orofacial behaviors including swallowing, vocalization and autonomic regulation, it is not surprising that many neuromodulators influence the breathing motor pattern through their actions on the brainstem respiratory network including, but not limited to serotonin, dopamine, acetylcholine, opioids, histamine, substance P and somatostatin (Doi and Ramirez, 2008; Lalley, 2008; Richter et al., 2003; Ashhad et al., 2022). Neurons which express pre- and post-synaptic receptors for neuromodulatory neurotransmission are highly distributed across the respiratory network. However, the effects of neuromodulation on breathing are commonly investigated at either a coarse scale by examining their effects on the frequency and amplitude of respiratory motor nerve activities after systemic drug application or at a finer scale via drug micro-injection within a particular compartment of the respiratory network. Consequently, the mechanisms of respiratory neuromodulation identified by these experimental approaches have highlighted the role of neuromodulation within single network compartments, especially the pre-Bötzinger complex (Doi and Ramirez, 2008; Ashhad et al., 2022), as the primary targets of neuromodulators. However, these studies do not consider the pattern of neuromodulatory neurotransmission across the entire network, which was a major aim of the present study.

The conundrum of neuromodulation is highlighted by research concerned with opioid-induced respiratory depression (ORID) evoked by overdose of opioid-based analgesics or drugs of abuse that predominantly bind to the *μ*-opioid receptor (μ-OR) (Lalley et al., 2014; Montandon and Horner, 2014; Ramirez et al., 2021). Mechanistically, μ-OR agonists have been shown to act on the medullary pre-BötC (Bachmutsky et al., 2020; Gray et al., 1999; Janczewski et al., 2002), ventral respiratory group (Ballanyi, 2004; Haji et al., 2003; Lalley, 2003; Rondouin, 1981; Takeda et al., 2001), and pontine parabrachial and Kölliker-Fuse nuclei (Eguchi et al., 1987; Hurlé et al., 1985; Mustapic et al., 2010; Prkic et al., 2012; Saunders et al., 2022; Varga et al., 2020). In addition to these functionally identified areas, a recent anatomical study of *Oprm1* expression in the respiratory network has identified μ-OR^+^ neurons in the nucleus tractus solitarii, Bötzinger complex, intermediate reticular nucleus/post-inspiratory complex, parafacial area, locus coeruleus and raphé nuclei (Furdui et al., 2024) suggesting that opioids may act simultaneously at many diverse sites across the brainstem respiratory network. Despite the widespread expression of μ-ORs, several studies have proposed that OIRD depends solely on the activation of μ-ORs in the pre-BötC to suppress inspiratory rhythm generation (Montandon and Horner, 2014; Bachmutsky et al., 2020; Montandon et al., 2011). Other studies have taken a more holistic view acknowledging the role of a distributed network mechanism for OIRD, but highlight the Kölliker-Fuse nuclei as a primary therapeutic target for OIRD (Lalley et al., 2014; Mustapic et al., 2010; Prkic et al., 2012; Varga et al., 2020).

This on-going debate has motivated the need to develop an understanding of the network mechanism of OIRD (Ramirez et al., 2021), and of respiratory network neuromodulation, in general. However, understanding the network mechanisms for neuromodulation in a distributed brain circuit would require defining not only the complete connectome of the circuit, but also the pattern of neuromodulatory co-transmitters and receptors expression across that connectome (Marder, 2012; Brezina, 2010). Here, to overcome this challenge, we combine computational modeling with ensemble electrophysiology to test the hypothesis that neuromodulatory systems in the respiratory network are organized to contribute to the maintenance of the breathing rhythm and pattern. To encapsulate this hypothesis, we follow the approach of Kleinfeld and Sompolinsky (1988) who developed a pair of Hebbian learning rules for the fast- and slow-synapses of a Hopfield network that produce the periodic sequential activities observed in central pattern generating networks. By training such a model to produce the respiratory firing patterns observed in the pre-Bötzinger complex (pre-BötC), an essential node of the respiratory network that expresses a representative set of firing patterns associated with all three phases of the breathing pattern under intact network conditions *in vivo* (Connelly et al., 1992; Schwarzacher et al., 1995; Sun et al., 1998) and *in situ* (St-John et al., 2009), we develop a model of the respiratory network in which the asymmetric pattern of slow-/neuromodulatory-connectivity drives the respiratory rhythm and pattern. To test our hypothesis, we compare the *in silico* simulations of increasing the strength of subsets of neuromodulatory connections based on the net effect on their post-synaptic targets with electrophysiologic experiments *in situ* in which we used a high-density multi-electrode array to monitor the ensemble activities of pre-BötC neurons before and after systemic administration of either the G_i/o_-coupled μ-OR agonist fentanyl or the G_i/o_-coupled 5HT1A receptor agonist 8-OH DPAT. In either case, we observed qualitatively similar responses of network activity to the perturbations in both simulations and experiments. Interestingly, the model also suggested the existence of a population code in which network activity is maximal at the transitions between the three phases of the breathing pattern, which we also observed experimentally in the ensemble activity of the pre-BötC. Taken together, we propose that neuromodulatory systems of the respiratory network are organized asymmetrically to contribute to the maintenance of the breathing rhythm and pattern. Furthermore, we conclude that activation of μ-ORs disrupts a network mechanism of respiratory rhythm and pattern generation.

## Materials and methods

Experimental protocols were approved by and conducted with strict adherence to the guidelines established by the Animal Ethics Committee of the Florey Department of Neuroscience and Mental Health, University of Melbourne, Melbourne, Australia (AEC No.: 17-075-FINMH). For breeding, adult male and female Sprague–Dawley rats (Animal Resources Centre, Canning Vale, Australia) and their offspring were housed under a 14:10 light/dark cycle with ad libitum access to standard laboratory chow and water.

### *In situ* arterially-perfused brainstem preparation

Experiments were performed in juvenile (17–26 days post-natal) Sprague–Dawley rats of either sex using the *in situ* arterially-perfused brainstem preparation as described previously (Paton, 1996; Paton et al., 2022). Briefly, rats were anesthetized by inhalation of isoflurane (2–5%) until they reached a surgical plane of anesthesia. Next, rats were transected sub-diaphragmatically and immediately transferred to an ice-cold bath of artificial cerebrospinal fluid (aCSF; in mM: [NaCl] 125, [KCl] 3, [KH_2_PO_4_] 1.25, [MgSO_4_] 1.25, [NaHCO_3_] 24, [CaCl_2_] 2.5 and [D-glucose] 10) for decerebration. Next, the heart and lungs were removed. The phrenic nerve was isolated for recording, and the descending aorta was isolated for cannulation. Next, the cerebellum was removed. Finally, the vagus and hypoglossal nerves were isolated for recording.

The preparation was then transferred to a recording chamber. The aorta was quickly cannulated with a double-lumen catheter. The preparation was then re-perfused with carbogenated (95%/5% pO_2_/pCO_2_), heated (31°C) aCSF (200 mL) using a peristaltic pump (Watson-Marlow).

Phrenic, vagal and hypoglossal nerves were mounted on suction electrodes to record the fictive respiratory motor pattern. Motor nerve potentials were amplified (400×), filtered (1–7,500 Hz), digitized (30 kHz) via a 16-channel differential headstage (Intan RHD2216), and stored on an acquisition computer using an Open-Ephys acquisition system [Rev. 2, (Siegle et al., 2017)]. Within minutes, apneustic respiratory contractions resumed.

The perfusion flow rate was adjusted to fine tune the preparation to generate a stable rhythm with augmenting inspiratory phrenic discharge and bi-phasic inspiratory and post-inspiratory activity on the vagus nerve. Finally, a single bolus of vecuronium bromide (0.3 mL, 0.1 mg/mL w/v vecuronium bromide: saline) was delivered to the perfusate to paralyze the preparation to avoid movement artifacts.

### Ensemble recording of pre-Bötzinger complex

In one series of experiments (*n* = 11), we measured single unit activities across ensembles of pre-BötC neurons using a 4-shank, 64-channel high-density silicon MEA (Neuronexus, A4x16) while simultaneously recording the three-phase respiratory motor pattern on phrenic, vagal and hypoglossal nerves. The MEA electrode sites spanned 345 μm in the dorso-ventral axis, and 600 μm in the rostro-caudal axis.

Using a micro-manipulator (Narishige MMN-33), we slowly inserted the MEA into the brainstem until we observed an ensemble of neuronal activities with respiratory-related firing patterns. The coordinates of the recording sites were measured from the caudal-most shank relative to those of calamus scriptoriius and were: 1.6–2.3 mm rostral to calamus scriptoriius, 1.4–1.8 mm lateral to the midline and 1.6–2.2 mm below the brainstem surface. Once positioned within the pre-BötC, we recorded the spontaneous activity of the pre-BötC ensemble for 10 min. Neuronal activities from the MEA were amplified (400×), filtered (0.001–7.5 kHz) and digitized via a 64-channel mono-polar headstage (Intan RHD2164) and stored on an acquisition computer using an Open-Ephys acquisition system.

**Figure 1:**
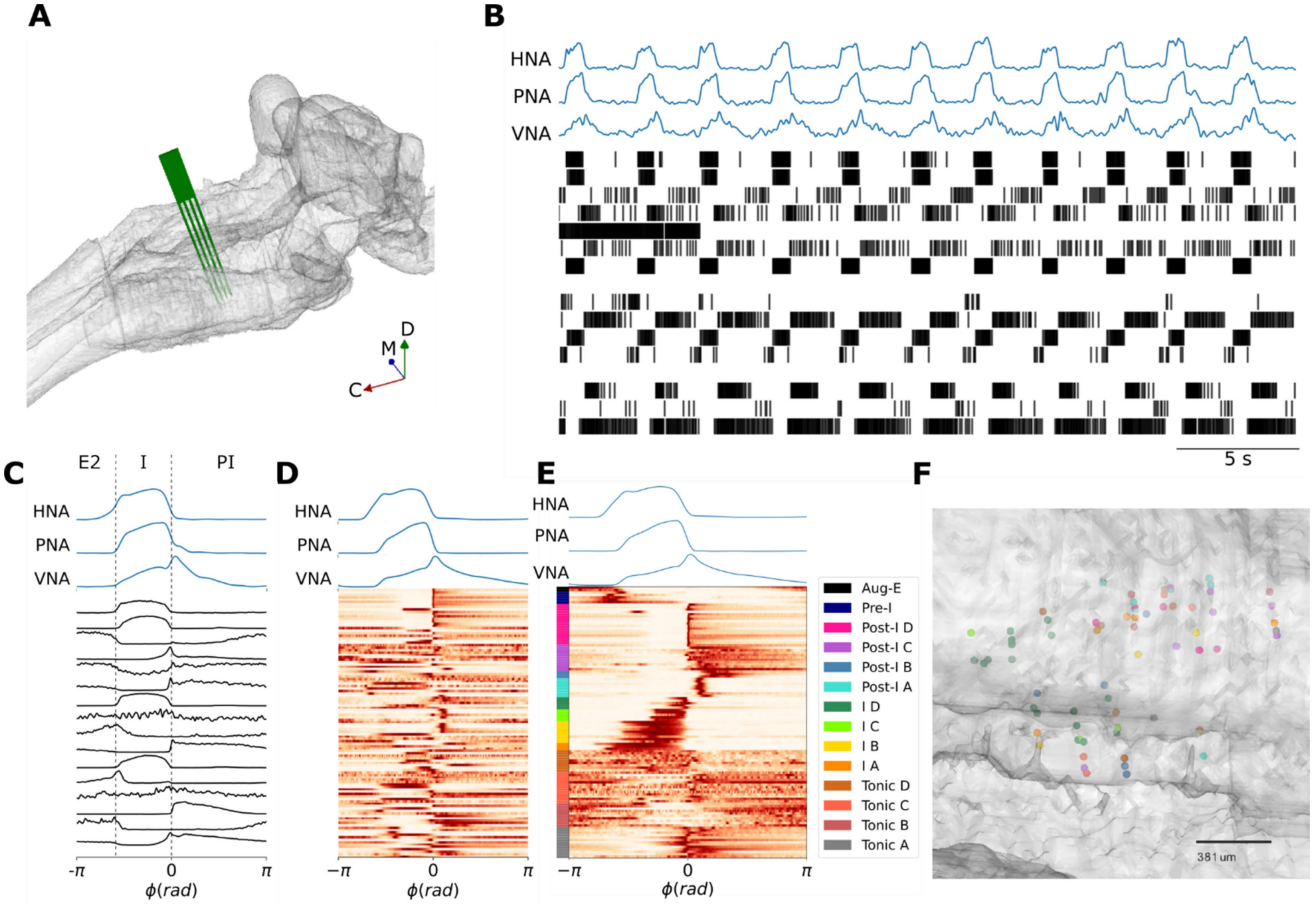
Identifying the pre-BötC neuronal firing patterns that underlie the three-phase respiratory motor pattern. **(A)** Reconstruction of an MEA positioned in the pre-BötC *in situ* in a representative experiment. **(B)** A representative recording of the three-phase respiratory motor pattern on hypoglossal (HNA), phrenic (PNA) and vagal (VNA) nerves in concert with an ensemble of pre-BötC neurons. **(C)** Cycle-triggered histograms of the pre-BötC neurons shown in panel **(A)** include neurons that spike in late-expiration (E2), inspiration (I) and post-inspiration (PI). *ϕ*: phase; rad: radians. **(D)** Cycle-triggered histograms of all recorded pre-BötC neurons before clustering. **(E)** K-means clustering identifies 14 classes of pre-BötC firing patterns that underlie the three-phase respiratory motor pattern. **(F)** Reconstructed locations of a subset of neurons suggest that pre-BötC neuronal types are spatially mixed. The rostral direction is to the right of the plot. μm: micrometers.

In a subset of these experiments, to enable mapping the location of pre-BötC neurons to the 7 T MRI Waxholm atlas of the Sprague–Dawley rat brain (Papp et al., 2014) by determining the rigid transformation necessary to register the positioner coordinate-system with those of the Waxholm atlas, we measured the coordinates of 5 surface landmarks that were easily identifiable both on the brainstem surface of the preparation and within the atlas (Supplementary Figure 1; Figures 1A,F). There was a maximal variation in the estimated rostro-caudal locations of the recorded neurons of 944 μm. Therefore, while we are confident that some MEA shanks in each experiment were positioned within the pre-BötC, it is likely that some of the recorded neurons were located in the adjacent rostral ventral respiratory group or BötC.

### Pharmacologic experiments

In subsequent experiments, to assess the effects of increasing neuromodulatory tone, after positioning the MEA within the pre-BötC and recording the stationary baseline pattern of pre-BötC ensemble activity, we administered either the 5HT1aR agonist 8-OH DPAT (1 μM, *n* = 4) or the μ-opioid receptor agonist fentanyl (15 nM, *n* = 8) to the perfusate and recorded the ensemble activity of the pre-BötC for an additional 10 min once the preparation expressed a new stationary breathing pattern (≤5 min).

### Data analysis

Phrenic, vagal and hypoglossal nerve activities (PNA, VNA and HNA, respectively) were first high-pass filtered with a zero-phase 3rd order Butterworth filter (*F_C_* = 300 Hz) to remove any DC artifacts before rectification and integration with a moving average filter in forward-backward mode to prevent any phase distortion (*τ* = 100 ms). The Kilosort algorithm was used for semi-automated spike sorting of single unit activities recorded on the MEA (Pachitariu et al., 2016). After spike sorting, we manually inspected and adjusted the cluster assignments. The most frequent modification made to cluster assignments was to remove low-amplitude clusters that were associated with noise or multi-unit activity.

After spike sorting, we sought to assess the distribution of pre-BötC neuronal firing patterns by clustering their respiratory cycle-triggered histograms. We first determined the event times of the inspiratory-to-post-inspiratory (I-PI) phase transition for all breaths via PNA. Depending on the signal-noise ratio of the PNA time series, we used either fixed threshold or the difference between a fast- (*τ* = 33.3 ms) and slow- (*τ* = 100 ms) moving averages to detect the I-PI transition events. After measuring the average respiratory period, we computed the cycle-triggered average of the respiratory motor pattern and the cycle-triggered histogram of each neurons spiking pattern over one respiratory cycle using the I-PI transition events as the trigger for averaging.

To cluster these cycle-triggered histograms of pre-BötC neuronal firing patterns for the group, we combined dimensionality reduction with principal component analysis (PCA) and k-means clustering. The cycle-triggered histograms were scaled to the [0, 1] interval to remove the influence of the peak firing frequencies. Then, we reduced the dimensionality of the scaled group dataset using PCA keeping the top principal components which accounted for >90% of the variance of the original dataset. The inverse transform of these top principal components further illustrated that no meaningful information was lost by discarding the remaining principal components (Supplementary Figure 2). Then, we determined the optimal number of clusters using the “elbow method”. To visualize the efficacy of the clustering, we examined scaled firing patterns sorted by the k-means cluster labels and used a t-Stochastic Neighbor Embedding to project the dimensionally reduced dataset (and k-means labels) into a 2-dimensional sub-space. Finally, to visualize the firing patterns of each cluster in the respiratory phase domain, we applied the inverse transform of the PCA to each k-means cluster center.

### Population coding and cross-correlation analyses

We first computed the firing rate histogram of each unit in a pre-BötC ensemble recording using a fixed bin width of 50 ms. The population rate time histogram was determined for each ensemble by taking the sum of the spike counts of all neurons in an ensemble for all bins (bin width: 50 ms) before converting the population spike counts into spike frequency. Both the individual pre-BötC firing rate histograms and the population rate were then smoothed with a 2nd order Savitsky-Golay filter with a window length of 5 bins. We measured the Pearson cross-correlation coefficient between VNA and the population firing rate. We chose VNA as an index of the three-phase respiratory motor pattern because its pattern reflects all three phases of the respiratory cycle. To assess the significance of this cross-correlation, we generated a bootstrap dataset (*n* = 500) in which we shuffled the inter-spike intervals of each unit before computing the population firing rate of the shuffled ensemble and measuring its correlation with VNA (see Supplementary Figure 11). To further characterize the relative timing between population firing rate peaks and the respiratory motor pattern, we measured the relative time difference between the I-PI transition and the nearest population firing rate peak. Finally, we examined the cycle-triggered averaged respiratory LFP in relation to the respiratory motor pattern and population firing rate.

### Pharmacologic experiments

In all pharmacologic experiments, we spike sorted 10 min of pre-BötC ensemble activity before and after drug administration. In experiments with 8-OH DPAT, neuronal activity was aligned according to the spike templates identified by the Kilosort algorithm. The significance of the increase in respiratory rate was determined using a one-way ANOVA. To assess the effect of 8-OH DPAT on the distribution of pre-BötC firing patterns, the cycle-triggered histograms of all neurons both at baseline and after drug administration were clustered as described above. The distributions of pre-BötC firing patterns were then compared using Fisher’s exact test. Because fentanyl evoked opioid-induced persistent apnea during which PNA was silent, we did not have any events to compute respiratory cycle-triggered averages, and hence could not apply dimensionality reduction and k-means clustering to characterize the distribution of neuronal firing patterns after fentanyl exposure. Thus, in fentanyl experiments, after spike sorting, we used the logISI method to identify bursts and burst-related spikes (Pasquale et al., 2010). To permit a better comparison with the simulations, we further sub-divided bursting neurons after fentanyl exposure into fast- and slow-bursting populations according to the median of their inter-burst interval. Once identified, we compared the inter-burst intervals and spikes/burst of baseline bursting, fentanyl-evoked fast- and slow-bursting populations using a one-way ANOVA.

### A Hopfield network model of respiratory pattern generation

We modeled the respiratory pattern generator as a Hopfield network that included fast- and slow-synapses. An all-to-all connected network of *N* = 70 discrete Hopfield neurons was trained via Hebbian learning rules for fast- and slow-synapses to generate a sequential, cyclical pattern of spiking in which various populations were active or silent (Kleinfeld and Sompolinsky, 1988; Hopfield, 1982; Kleinfeld and Sompolinsky, 1989).

### Network dynamics

Following (Kleinfeld and Sompolinsky, 1988; Kleinfeld and Sompolinsky, 1989), the output of the *i*th neuron, $V_{i}(t)$ is related to its net synaptic input $u_{i}(t)$ by a gain function *g*[*x*]:


(1) $$V_{i}(t)=g[u_{i}(t)-\theta_{i}]$$

We modeled the neuronal dynamics in the high-gain limit where *g*[*x*] is just the Heaviside step function such that:


(2) $$V_{i}(t)=\{\begin{array}{c} 1,u_{i}(t)>\theta_{i}\\ 0,u_{i}(t)\leq \theta_{i} \end{array}\;\;$$

The Hopfield neurons in the model included both fast- and slow-synaptic inputs, $h_{i}^{F}$ and $h_{i}^{S}$ respectively. The net synaptic input to the *i*th neuron, $u_{i}(t)$, is:


(3) $$\begin{array}{c} \tau_{F}\frac{{\mathrm{du}}_{i}(t)}{\mathrm{dt}}+u_{i}(t)=h_{i}^{F}(t)+h_{i}^{S}(t)\\ =\sum_{j=1}^{N}[w_{\mathrm{ij}}^{F}V_{j}(t)+w_{\mathrm{ij}}^{S}\;\bar{V_{j}(t)}] \end{array}$$

where $\tau_{F}$ is the time-constant of the fast synapses, $w_{\mathrm{ij}}^{F}$ and $w_{\mathrm{ij}}^{S}$ are the synaptic weights of the fast and slow synapses, respectively, and $\bar{V_{j}(t)}$ is the time-averaged output of the neuron, i.e.:


(4) $$\bar{V_{j}(t)}=\int_{0}^{\infty }V_{j}(t-t\prime )w(t\prime )\mathrm{dt}\prime$$

The synaptic response function *w*(*t*) for the slow weights, $w_{\mathrm{ij}}^{S}$, is a non-negative function normalized to unity and characterized by a mean time constant $\tau_{s}$satisfying.


(5) $$\int_{0}^{\infty }\mathrm{tw}(t)\mathrm{dt}=\tau_{S}$$

The ratio between $\tau_{F}$ and $\tau_{S}$ determines the time spent in each state before a transition occurs (Kleinfeld and Sompolinsky, 1988; Kleinfeld and Sompolinsky, 1989). The effects of changing this ratio are shown in Supplementary Figures 4, 5. In our model, $\tau_{F}$ and $\tau_{S}$ were 5 and 10 timesteps, respectively.

### Hebbian learning of respiratory spiking patterns

The network was trained via Hebbian learning rules to oscillate through a set of states, ${\left\{S^{\mu }\right\}}_{\mu =1}^{r}$, that are each defined by the activity (high-frequency spiking or silent/low-frequency spiking) of all *N* neurons, and that cyclically progress through their defined sequence


(6) $$S^{1}\to S^{2}\to \ldots \to S^{r-1}\to S^{r}\to S^{1}$$

**Figure 2:**
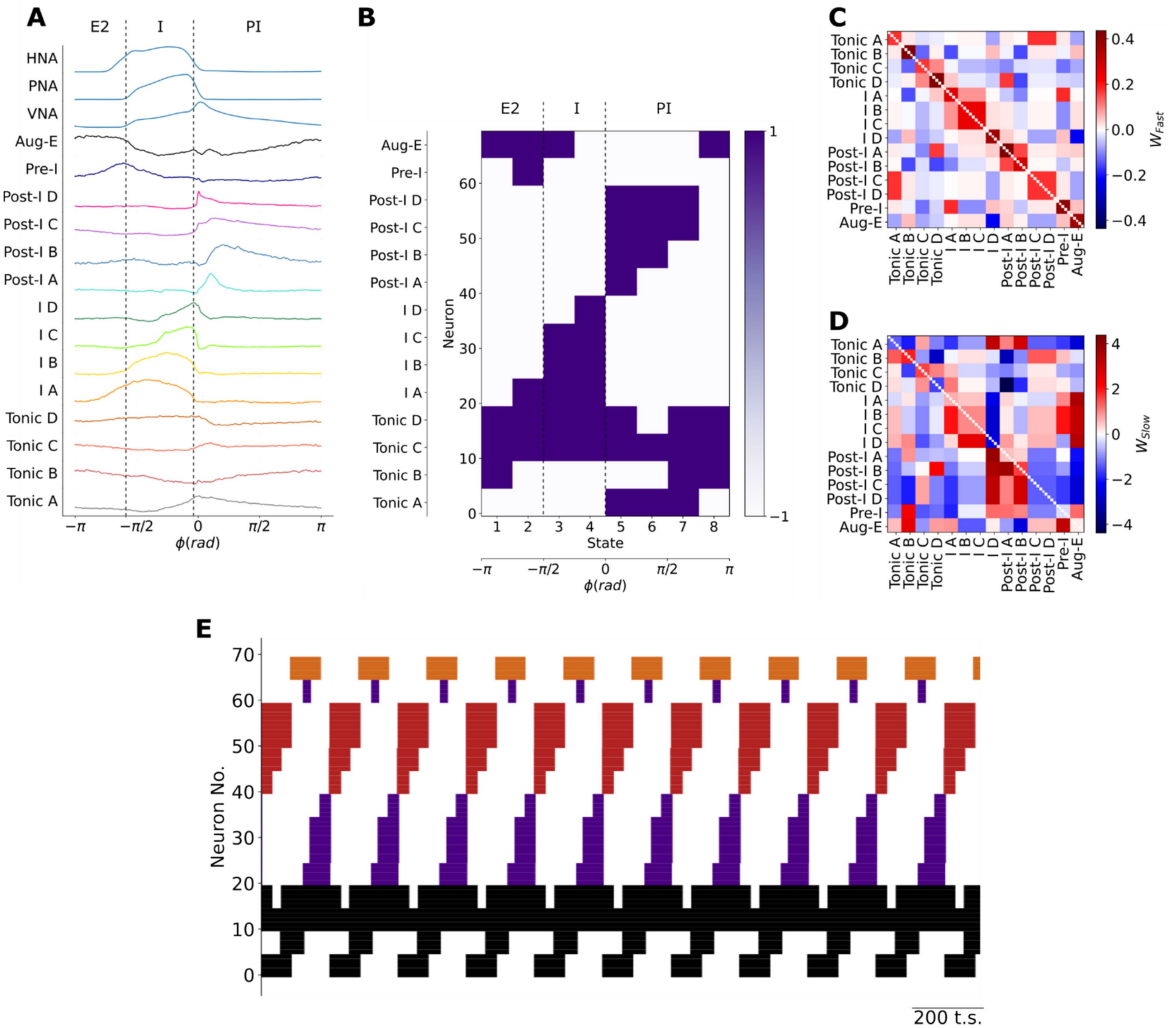
Training a Hopfield model to encode the firing patterns of pre-BötC neuronal clusters. **(A)** The centroids of pre-BötC neuronal clusters were used as a basis to determine the sequential firing patterns to be encoded in the model. The respiratory cycle was discretized into eight sequential states to account for the brief firing patterns of the Pre-I, Post-I B and I-D populations. I: inspiratory; E: expiratory. **(B)** Each pre-BötC cluster was represented by 5 neurons in the model. Their training vectors were taken as +1 when the cluster fired at a high-frequency or −1 when the cluster was silent or firing at a low-frequency. **(C,D)** The resultant fast- **(C)** and slow- **(D)** synaptic connectivity of the Hopfield network after training to encode the sequential state vectors using Hebbian learning rules. $w_{\text{Fast}}$: fast synaptic weight; $w_{\text{Slow}}$: slow synaptic weight. **(E)** As expected, the model generated the learned sequential firing patterns that underlie the three-phase respiratory motor pattern in the pre-BötC. Black: tonic or respiratory-modulated; Purple: inspiratory; Red: post-inspiratory; Orange: late-expiratory; Neuron No.: Neuron number; t.s.: timesteps.

The pre-BötC respiratory firing patterns were not orthogonal (see Figure 2B for states sequence), and therefore, we followed the method proposed by Kleinfeld and Sompolinsky (1989) to encode these non-orthogonal states in the network. We first define the correlation matrix of states as:


(7) $$C_{\mu ,\mu +1}=\frac{1}{N}\sum_{i=1}^{N}S_{i}^{\mu }S_{i}^{\mu +1},\forall \mu =1,\ldots ,r$$

Then, orthogonal states can be constructed from linear combinations of the


$${S^{\mu }}_{s}$$

(8) $$O_{i}^{\mu }=\sum_{\mu =1}^{r}C_{\mu ,\mu +1}^{-1}S_{i}^{\mu +1}$$

where $C_{\mu ,\mu +1}^{-1}$ is the pseudo-inverse of the correlation matrix of states.

Finally, the network is trained using the following equations to determine the weights of the fast- and slow-synapses, respectively.


(9) $$w_{\mathrm{ij}}^{F}=\frac{J_{0}}{N}\sum_{\mu =1}^{r}S_{i}^{\mu }O_{j}^{\mu },\forall i\neq j$$

(10) $$w_{\mathrm{ij}}^{S}=\lambda \frac{J_{0}}{N}\sum_{\mu =1}^{r-1}S_{i}^{\mu +1}O_{j}^{\mu },\forall i\neq j$$

where $J_{0}/N$ sets the scale of the average synaptic strength (Supplementary Figures 6, 7) and *λ* is a parameter that determines the transition strength between successive states. For all models shown, $J_{0}$ was 3, N was 70 (i.e., 5 neurons/class) and *λ* was 10.

### Simulations of increasing slow neurotransmission

To simulate the effects of application of a neuromodulatory receptor agonist, we modeled the effects of increasing either slow-inhibition or -excitation in the model by increasing the weights of these synapses by 1.5× their original magnitude. Because endogenous neuromodulators have been shown to continually influence respiratory network activity (Doi and Ramirez, 2008; Shao and Feldman, 2005), we considered that perturbations (increasing or decreasing) of the weights of slow synapses in the model in a narrow range around its original value would be comparable to the experimental perturbation of systemically applying neuromodulatory receptor agonists or antagonists.

Unless stated otherwise, measurements are reported as means ± standard deviation.

## Results

### Training a Hopfield network to generate the breathing pattern

Recurrent Hopfield networks that generate periodic activities can be trained via Hebbian learning rules given the rhythmic firing patterns of the network (Kleinfeld and Sompolinsky, 1988; Kleinfeld and Sompolinsky, 1989). Therefore, we first needed to estimate the set of respiratory neuron firing patterns in the intact network of the *in situ* perfused preparation and chose to do so in the pre-BötC since it contains neurons from all three phases of the breathing pattern *in vivo* (Connelly et al., 1992; Schwarzacher et al., 1995; Sun et al., 1998).

To accomplish this, we used a high-density silicon MEA to monitor ensemble single-unit activities of the pre-BötC in concert with the respiratory motor pattern on phrenic (PNA), vagal (VNA) and hypoglossal (HNA) motor nerves in the *in situ* perfused brainstem preparation (Figures 1A,B). We clustered the cycle-triggered histograms of their activity using the transition from inspiration to post-inspiration (I-PI transition) as the trigger for averaging across one respiratory cycle (Figures 1C–E). Cycle-triggered histograms were determined for 113 neurons from 11 *in situ* preparations (Figure 1D). To optimize the sensitivity of the clustering to the firing patterns of pre-BötC neurons, the dataset was further pre-processed by scaling to the [0, 1] interval to eliminate the influence of firing rate variability, and by using a principal component analysis (PCA) for dimensionality reduction. After pre-processing, the dataset was clustered using the K-means algorithm (Figure 1E). The optimal number of pre-BötC neuronal types (k* = 14) was determined using the ‘elbow method’ after repeating the K-means clustering for many values of k.

The pre-BötC of rats *in situ* displayed a mixture of inspiratory, post-inspiratory, late-expiratory and phase-spanning firing patterns (Figure 1E). As the purpose of our clustering analysis was to develop a consistent, un-biased assessment of the diversity of pre-BötC neuronal types, we avoid introducing a new nomenclature, and instead label the clusters according to a phenotypic division of the classical respiratory neuron types that are often used in central pattern generator models of respiratory pattern generation: pre-inspiratory, inspiratory, post-inspiratory, augmenting-expiratory or tonic-respiratory-modulated. The clustering analysis revealed that of these principal pre-BötC neuronal classes, inspiratory, post-inspiratory and tonic pre-BötC neurons had distinct sub-classes (Figure 1E). For instance, the clustering analysis revealed that post-inspiratory pre-BötC neurons were further sub-divided into 4 sub-classes (see clusters Post-I A-D, Figure 1E) that differed in their burst durations and the relative timing of their peak intra-burst frequencies. Finally, consistent with previous results (Connelly et al., 1992; Schwarzacher et al., 1995; Sun et al., 1998), we observed that the anatomic locations of pre-BötC neuronal types with distinct activity patterns across the respiratory cycle were spatially mixed (Figure 1F).

To construct the sequential states needed to train the model (see Materials and Methods: Hebbian learning of respiratory spiking patterns), we discretized the firing patterns of each pre-BötC neuronal type (Figure 2). The cluster centroids of each pre-BötC cluster were taken as the putative firing patterns of each neuronal type (Figure 2A). The model consists of a network of 70 Hopfield units with fast- and slow-synapses. Based on previous studies (Alheid and McCrimmon, 2008; Bush and Ramirez, 2024), we assumed that the measured distribution of respiratory firing patterns may have been biased by the locations of the recording site. Therefore, we represented each pre-BötC neuronal class by 5 model units. The respiratory cycle was sub-divided into 8 sequential states of *π*/4 radians to enable the approximation of the activity of very transiently active neuron populations like the Post-I B and I D clusters. For each cluster and for each fraction of the respiratory cycle, the state was taken as 1 when the cluster fired at a high frequency and −1 when the population was less active or silent (Figure 2B). Using these sequential state vectors, we trained the network to encode these sequential firing patterns using Hebbian learning rules. The resultant network weights are shown in Figures 2C,D. The fast-synapses had a symmetric structure consistent with their role in encoding the fixed points associated with each network state (Figure 2C), whereas the slow-synapses had an asymmetric structure consistent with their role in destabilizing any given fixed point in the direction of the next sequential fixed point (Figure 2D). As expected, the trained model generated these sequential firing patterns associated with the three-phase respiratory motor pattern in the absence of external input (Figure 2E), thereby fulfilling the definition of a central pattern generator network. Thus, we generated an associative memory network model of breathing pattern generation that was constrained by the representation of the three-phase respiratory motor pattern within the pre-BötC. We next validated the rhythmogenic role of the slow, neuromodulatory synapses with additional simulations and experiments.

### The role of neuromodulation in respiratory rhythm generation

**Figure 3:**
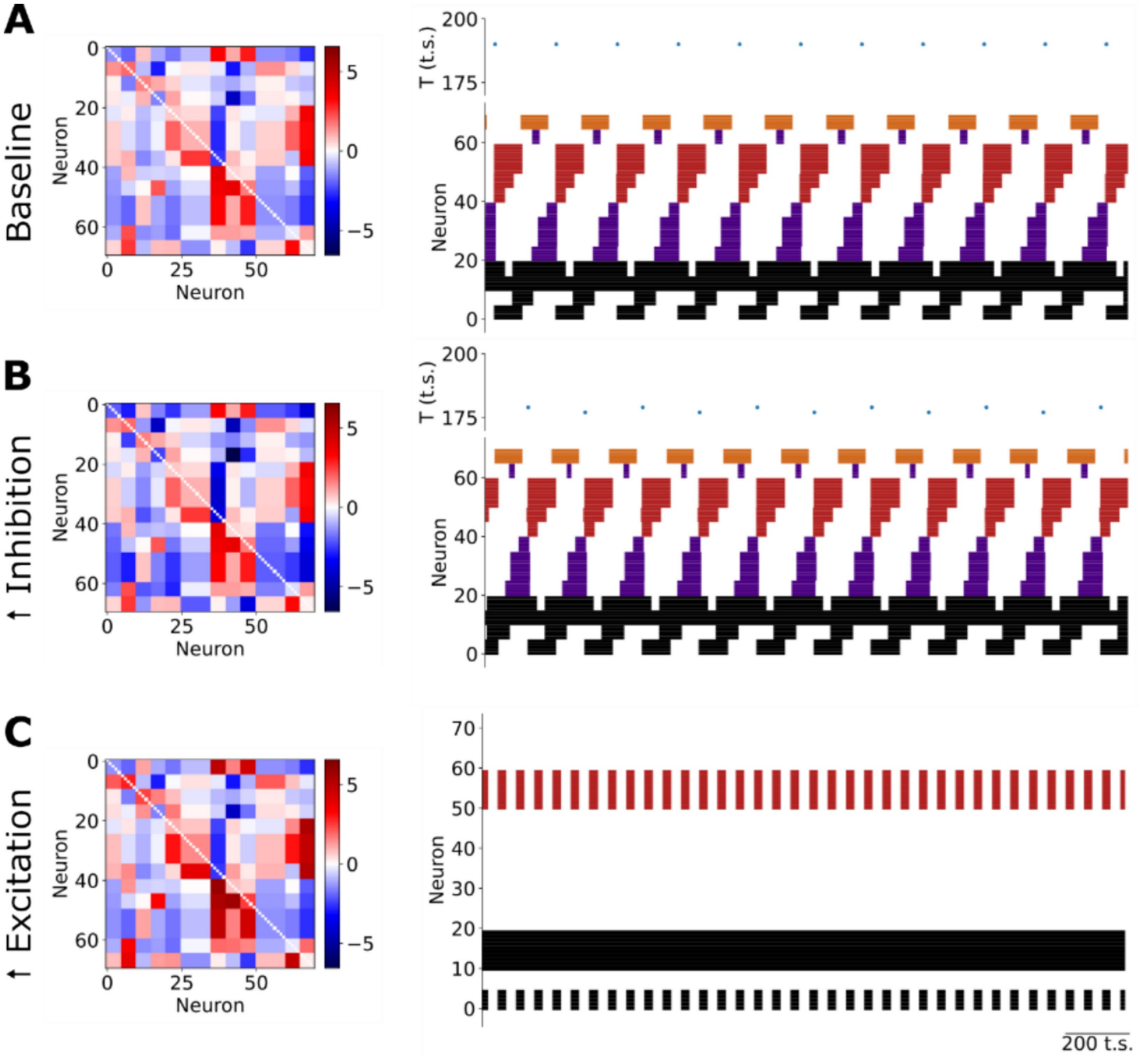
Neuromodulation of the respiratory rhythm *in silico*. **(A)** The sequential firing pattern produced by the network at baseline. T: period; t.s.: timesteps. **(B)** Increasing slow synaptic inhibition in the model evokes an increase in the frequency of the respiratory rhythm without any change in the sequential firing pattern of the network. T: period; t.s.: timesteps. **(C)** Increasing slow excitation in the model evokes a collapse of the respiratory rhythm characterized by a reduction in the number of active units whose remaining activity was either tonic or fast bursting. t.s.: timesteps.

**Figure 4:**
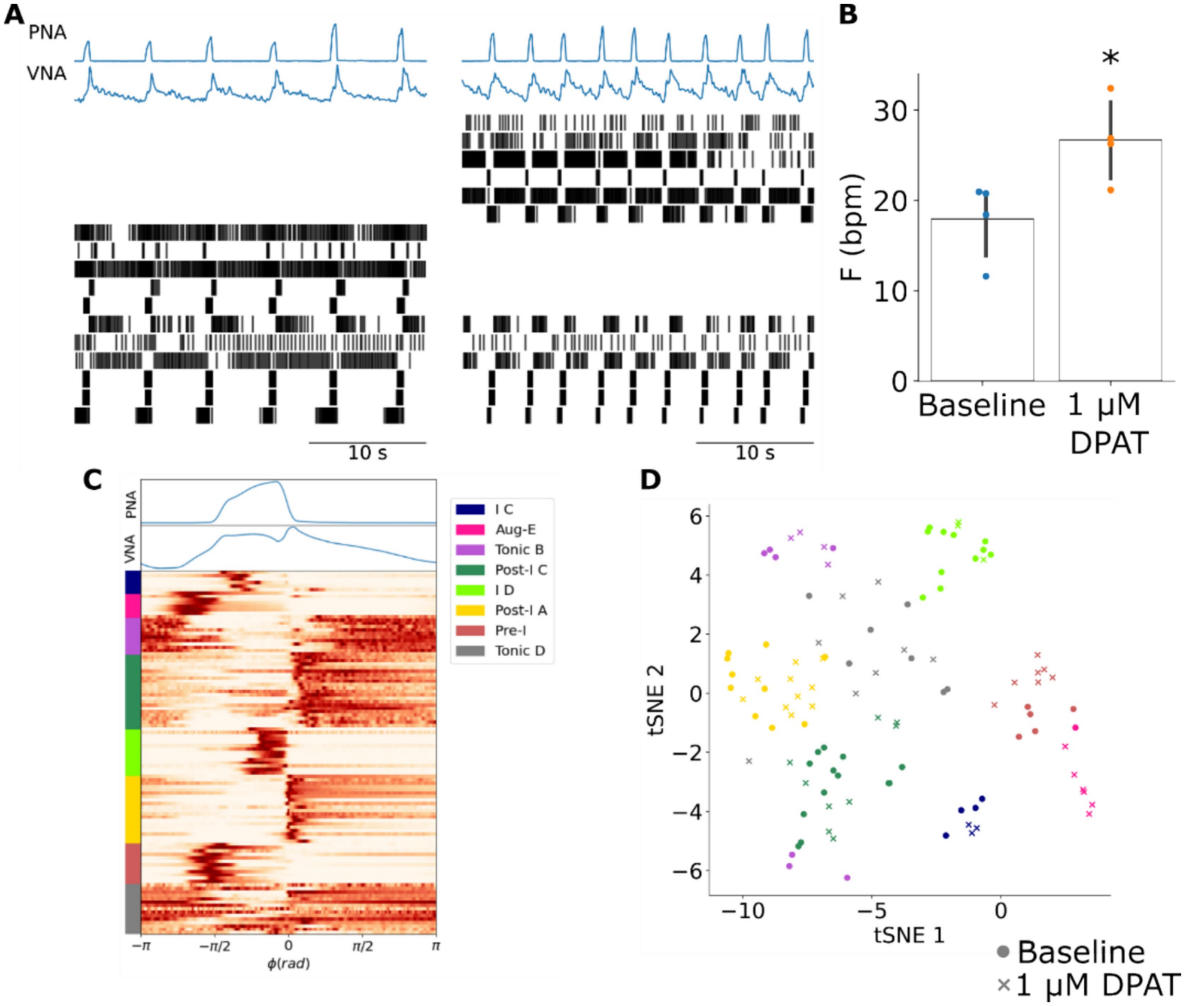
The 5HT1aR agonist 8-OH DPAT increases the frequency of the respiratory rhythm without changing the firing pattern of pre-BötC ensembles. **(A)** Systemic administration of 8-OH DPAT evoked an increase in the frequency of the respiratory rhythm as observed in PNA and VNA that was associated with a reconfiguration of pre-BötC ensemble activity. In this representative experiment six pre-BötC neurons maintained their original firing patterns, but at a higher frequency. In addition, five pre-BötC neurons became silent, and six pre-BötC neurons were activated. Left panel: baseline; right panel: after systemic 1 μM DPAT. **(B)** The frequency of the respiratory rhythm was significantly increased after systemic application of 8-OH DPAT. * *p* < 0.05. F: frequency; bpm: breaths per minute. **(C)** K-means clustering of all recorded pre-BötC neurons at baseline and after systemic 8-OH DPAT identified many of the pre-BötC neuronal types previously observed. *ϕ*: phase; rad: radians. **(D)** All clusters except the Aug-E population were present at similar ratios at baseline (circles) and after systemic 8-OH DPAT (crosses) suggesting that despite the reconfiguration of pre-BötC ensemble activity, the distribution of neuronal firing patterns that composed the respiratory pattern generator remained the same. Fisher’s exact test: *p* = 0.232. tSNE: t-Stochastic Neighbor Embedding.

**Figure 5:**
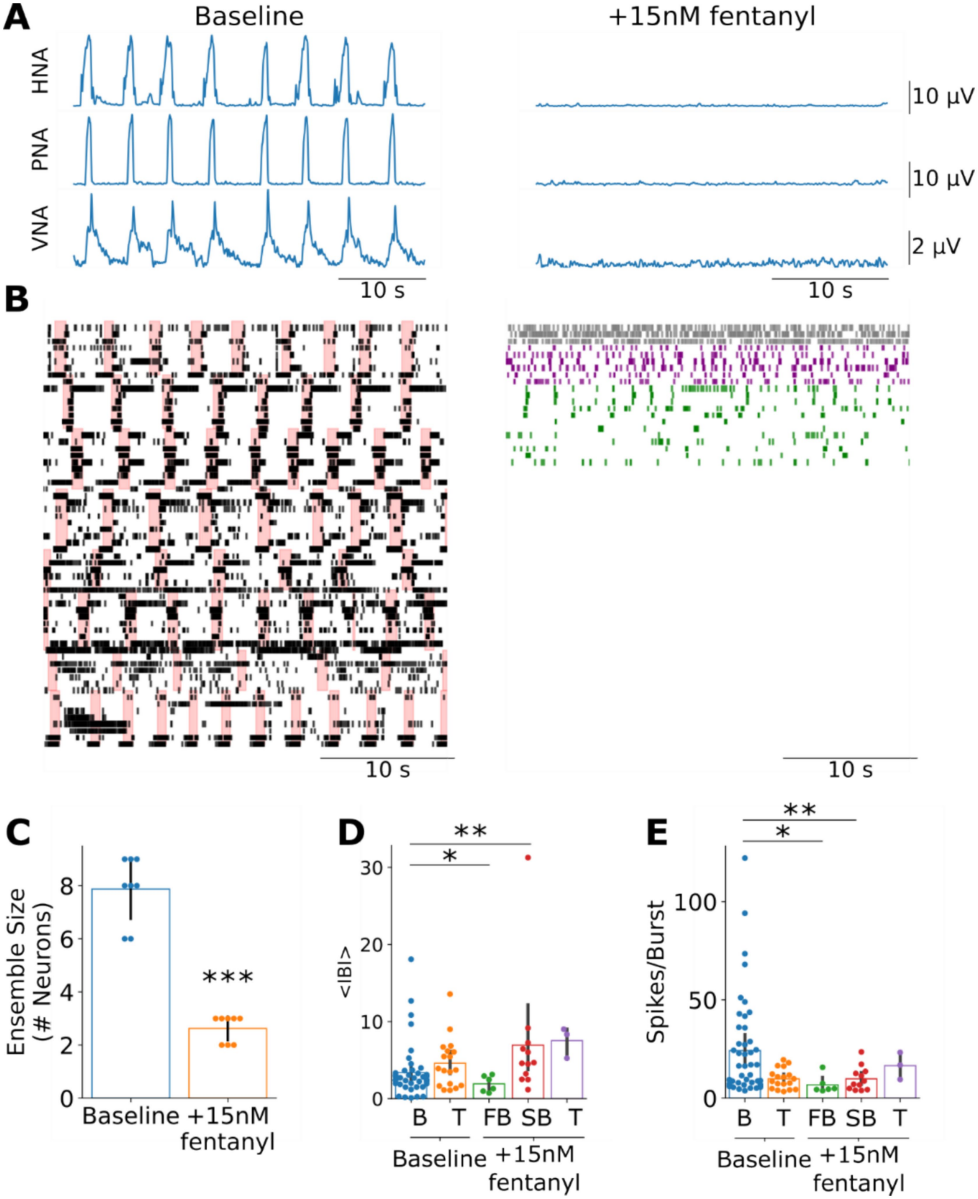
The reconfiguration of pre-BötC ensemble activity after opioid-induced respiratory depression is consistent with model simulations after increasing slow-excitation. **(A)** Systemic fentanyl administration evokes a collapse of the respiratory rhythm on phrenic (PNA), hypoglossal (HNA) and vagal (VNA) nerves. **(B)** Consistent with the model simulations of uniformly increasing slow excitation, systemic fentanyl administration was associated with a reduction in the size of pre-BötC ensembles and spared tonic (gray), fast- (purple) and slow- (green) bursting firing patterns. Please note that these raster plots reflect neuronal ensembles compiled from all experiments to better visualize the firing patterns expressed after fentanyl-evoked opioid-induced persistent apnea. As such, the ordering of units post-fentanyl exposure (right panel) do not have a one-to-one correspondance with the neuronal rasters at baseline. Further, to aid in the visualization of baseline firing patterns, we have plotted pink bars to indicate the inspiratory period for each pre-BötC ensemble. **(C)** Systemic fentanyl administration significantly reduced the number of active neurons in pre-BötC ensembles. ***p < 0.001. **(D)** Consistent with the model, fast-bursting pre-BötC neurons had significantly shorter inter-burst intervals (IBI in seconds) than bursting pre-BötC neurons at baseline. However, we also observed a slow-bursting pre-BötC population after systemic fentanyl administration that had significantly longer IBIs than bursting pre-BötC neurons at baseline. B: bursting; T: tonic; FB: fast-bursting; SB: slow-bursting *p < 0.05; **p < 0.01. **(E)** Consistent with the model, both fast- and slow-bursting pre-BötC neurons fired fewer spikes per burst than bursting pre-BötC neurons at baseline. *p < 0.05; **p < 0.01.

The model encapsulates our hypothesis that the asymmetric connectivity of slow-, neuromodulatory-synapses contributes to respiratory rhythm generation. Because the model contains slow-synapses that are described by their net excitatory or inhibitory effect on the post-synaptic target, we simulated the effects of uniformly increasing the weights of either slow-inhibitory or -excitatory synapses *in silico* (Figure 3). We chose to increase these slow synaptic weights to enable comparison with the experimental perturbation of systemic administration of neuromodulatory receptor agonists. Increasing the weights of slow-inhibitory synapses in the model evoked only minor effects on network activity (Figure 3B). Specifically, the sequential firing patterns of the network remained unchanged. However, the frequency of the network’s rhythm increased by 12%. The alternative perturbation, increasing the weights of slow-excitatory synapses, evoked a collapse of the respiratory rhythm (Figure 3C). With the increase in slow excitatory neuromodulation in the model, the majority of neurons (~64%) fell silent. The remaining active neurons expressed either tonic or bursting activities. The remaining bursting pattern of activity was characterized by shorter burst durations and inter-burst intervals than any bursting activity observed at baseline (compare Figures 3A,C). To validate these *in silico* observations experimentally, we analyzed the effect of systemic administration of either the 5HT1aR agonist 8-OH DPAT (Figure 4) or the *μ*-opioid receptor agonist fentanyl (Figure 5) on pre-BötC ensemble activity *in situ*.

Systemic application of 8-OH DPAT evoked effects on pre-BötC ensemble activity that were qualitatively similar to the effects of increasing slow inhibition in the model. 8-OH DPAT increased the frequency of the respiratory rhythm (Figures 4A,B). This increase in respiratory rate was accompanied by a reconfiguration of pre-BötC ensemble activity wherein some units became silent, previously silent units became active and some units maintained their baseline firing patterns, and a smaller subset of units changed their firing patterns (Figure 4A). To compare the experimental results with the corresponding simulation of increasing slow-inhibition, we assessed whether the distribution of respiratory neuron firing patterns was different from that under baseline conditions. To do so, we clustered the cycle-triggered histograms of all units before and after systemic 8-OH DPAT (Figure 4C). All firing pattern clusters contained units from both baseline and 8-OH DPAT groups (Figure 4D). Importantly, the distribution of pre-BötC firing patterns after systemic 8-OH DPAT was not significantly different from that at baseline (Supplementary Figure 9, *p* = 0.232) suggesting that despite the more complex reconfigurations that occurred in single trials, the overall distribution of pre-BötC firing patterns remained the same. Taken together, these results suggest that exogenous enhancement of 5HT1aR transmission evokes qualitatively similar effects as those evoked by an increase of slow inhibition in the model.

The experimentally observed effects of fentanyl on pre-BötC ensemble activity were qualitatively similar to of the effects of increasing slow excitation in the model. Systemic administration of 15 nM fentanyl evoked a collapse of the respiratory motor pattern on phrenic, vagal and hypoglossal nerves (Figure 5A). Consistent with the model, ensemble activity in the pre-BötC was largely suppressed with the number of active neurons from 7.8 ± 1.2 to 2.6 ± 0.5 neurons (Figures 5B,C, *p* < 0.001). Further, pre-BötC neuronal activity after systemic fentanyl administration consisted of neurons with either tonic or bursting activities. However, unlike the model, we observed that the distribution of the mean inter-burst intervals of bursting neurons post-fentanyl administration appeared bimodal (Supplementary Figure 10), with either fast-bursting and slow-bursting phenotypes, which were defined using a median threshold. Consistent with the model, both classes of bursting neurons had shorter burst durations than at baseline, firing significantly fewer spikes per burst (Figure 5E, Fast-Bursting: *p* < 0.05, Slow-Bursting: *p* < 0.01). Further, like the model simulations, the fast-bursting class also had significantly shorter inter-burst intervals than bursting pre-BötC neurons at baseline (Figure 5D, *p* < 0.05). However, the slow-bursting class had significantly longer inter-burst intervals than bursting pre-BötC neurons at baseline (Figure 5D, *p* < 0.01). Taken together, the effects of perturbing neuromodulatory transmission in the model were consistent with experimental results suggesting that neuromodulation may contribute to respiratory rhythm generation.

### Population activity encodes respiratory phase transitions

**Figure 6:**
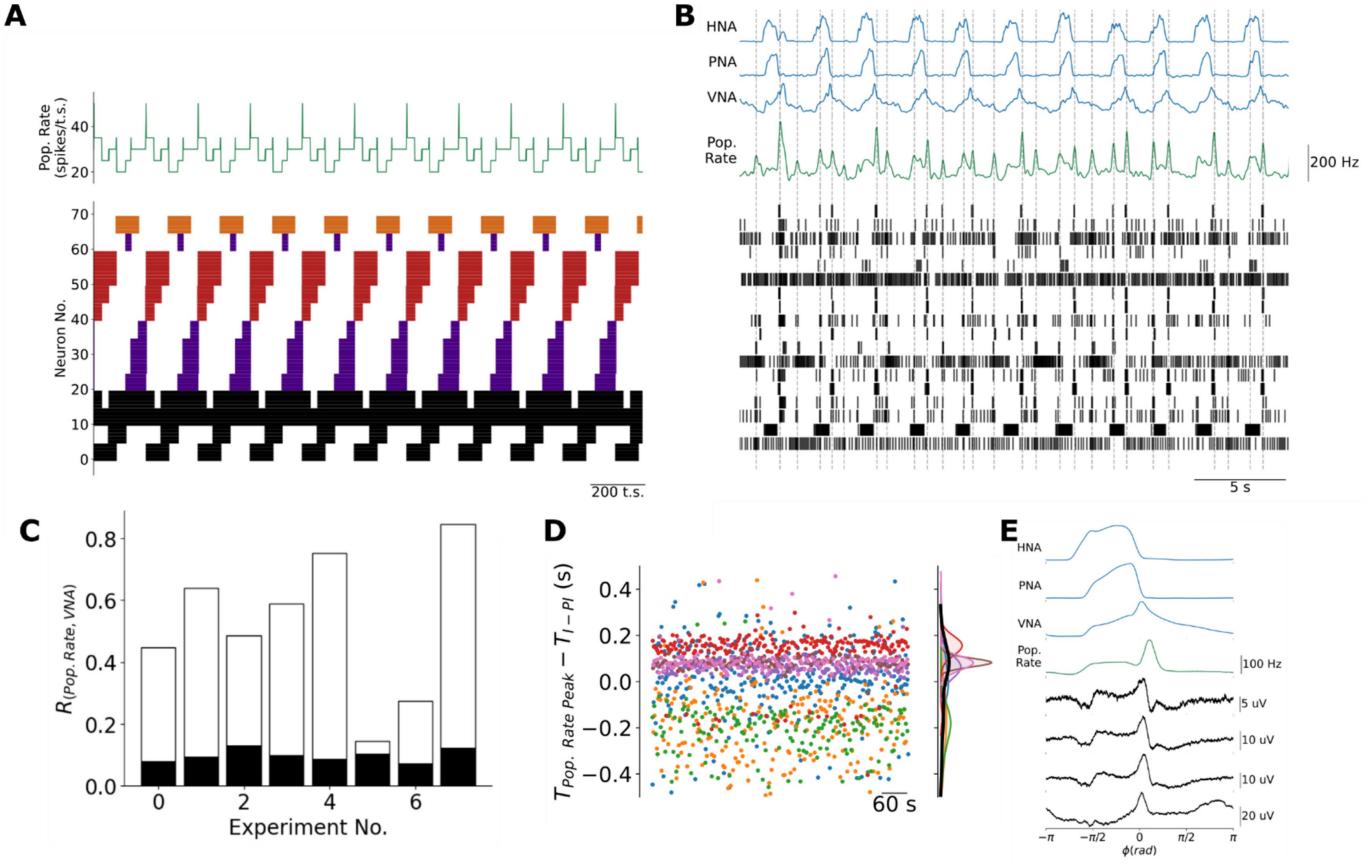
Population coding of the transitions between respiratory phases in the model and in the pre-BötC *in situ*. **(A)** Because the recall of the next sequential state involves a slight overlap of sequential assemblies, the model generates brief peaks in the population firing rate at each of the three transitions between respiratory phases when the adjacent state vectors are most distant. Black: tonic or respiratory-modulated; Purple: inspiratory; Red: post-inspiratory; Orange: late-expiratory; Pop. Rate: population firing rate in spikes/timestep. **(B)** Consistent with the model, pre-BötC ensemble activity is associated with a population firing rate (Pop. Rate, *green trace*) that also peaks at or near the transitions between respiratory phases. **(C)** There was a significant cross-correlation between the population firing rate and the three-phase respiratory pattern of vagal nerve activity (VNA). This cross-correlation was considered significant if it was greater than the 99.9%-ile of a bootstrap distribution in which the inter-spike intervals for each unit were shuffled before computing the shuffled ensemble’s population firing rate and its correlation with VNA. White bar: original cross-correlation between population firing rate and VNA; Black bar: upper bound of the 99.9%-ile of the bootstrap distribution. **(D)** Individual pre-BötC ensembles varied with respect to the precision with which their population firing rate encoded the I-PI transition. Please note that each color represents a different ensemble recorded in different preparations. $T_{\mathrm{Pop}.\text{RatePeak}}$: time of the peak in population rate in seconds; $T_{I-\mathrm{PI}}$: time of the transition from inspiration to post-inspiration, i.e., the time of the inspiratory off-switch, in seconds. **(E)** Cycle-triggered averages of pre-BötC local field potentials (LFPs) more reliably encoded the transitions between respiratory phases. Pop. Rate: Population firing rate.

Another feature of the model was the existence of a population code of the respiratory motor pattern (Figure 6). In the model, transitions between successive states occur because of the slow-synaptic neurotransmission that changes the energy landscape of the network. During any given state, the network lies at a global energy minimum leading to the repetitive firing of neurons associated with that fixed point. Because of the asymmetric connectivity of the slow synapses in the network, each fixed point is progressively destabilized until the fixed point associated with the next sequential state becomes the new global minimum. When this critical point is reached, the network rapidly transitions to the new fixed point thereby recalling the activity pattern of the next sequential state. These transitions are not instantaneous. Each state transition involves a short overlap of the activity patterns associated with the two successive states during this pattern recall process causing peaks in the population firing rate. We observed that the population activity of the network resembled the bi-phasic waveform expressed in vagal nerve activity, and that three of the eight state transitions—from I to PI, from PI to E2 and from E2 to I—were associated with brief peaks in population activity (Figure 6A, *top green trace*) when the distance between successive state vectors was maximal.

To test whether the intact respiratory network *in situ* also generates a population code of respiratory phase transitions, we measured the population firing rate of pre-BötC ensembles *in situ*. Like the model, the population activity of the pre-BötC ensembles was characterized by a basal level of activity interspersed with brief peaks of fast spiking (Figure 6B). Consistent with the model, the peaks in population activity occurred at or near the three transitions between respiratory phases. To assess this feature experimentally, we measured the cross-correlation between the population activity and the vagal motor pattern, which carries information about all three phases of the respiratory motor pattern (Figure 6C). This cross-correlation was always greater than the 99.9th percentile of a bootstrap distribution generated by shuffling the inter-spike intervals of each unit before computing the shuffled ensemble’s population firing rate and its correlation with VNA.

To further investigate whether the timing of pre-BötC population activity was specifically associated with the transitions between respiratory phases, we focused on the relative timing between ensemble population activity peaks that occurred nearest to the transition between I and PI (Figure 6D). On average, the ensemble population activity peak occurred 0.0024 ± 0.206 s before the decline in inspiratory PNA amplitude reflecting the fact that individual ensembles differed greatly with respect to the precision of and relative timing of their encoding the I-PI transition (Figure 6D). We hypothesized that this variability may be due to the limited number of pre-BötC neurons that we were able to simultaneously monitor using silicon MEAs. Therefore, we further addressed this question by measuring the cycle-triggered averages of respiratory local field potentials (LFPs) on each of the four MEA shanks and ensemble population activity in a representative experiment since LFPs reflect the synaptic activity of many more neurons (Figure 6E). Respiratory LFPs in the pre-BötC occurred specifically at the E2-I and I-PI transitions, whereas the ventral-most site of the fourth shank identified respiratory LFPs occurring specifically at the I-PI and PI-E2 transitions. Taken together, these data are consistent with the model in that population activity within the respiratory network peaks at the transitions between respiratory phases, a feature that is not present in the population activity of CPG models of respiratory pattern generation (Supplementary Figure 3).

## Discussion

In this study, we have developed a Hopfield network model of respiratory rhythm and pattern generation that encapsulates the hypothesis that slow-, neuromodulatory-connectivity in the respiratory network is organized asymmetrically to generate the respiratory rhythm. We tested this model assumption by comparing simulations of uniformly increasing slow-inhibitory or -excitatory weights with *in situ* experiments in which we recorded ensemble activity of the pre-BötC before and after systemic administration of 5HT1aR or μ-OR agonists. Increasing slow-inhibitory weights in the model or activating 5HT1aRs systemically with 8-OH DPAT increased the frequency of the respiratory rhythm without changing the firing patterns of respiratory neurons. Increasing slow-excitatory weights in the model or activating μ-ORs systemically with fentanyl arrested the respiratory rhythm sparing neurons with tonic- and short bursting-firing patterns. The similarity between model simulations and experiments supports the hypothesis that neuromodulatory connectivity in the respiratory network is organized asymmetrically to promote rhythmogenesis. The model also suggested the existence of a population code of respiratory phase transitions which we confirmed in the population activity of pre-BötC ensembles and respiratory LFPs in the pre-BötC.

### Network models of respiratory rhythm and pattern generation

Computational models of respiratory rhythm and pattern generation have been developed to explain experimental observations at both cellular and network scales. The discovery of spontaneously bursting pre-inspiratory neurons of the pre-BötC led to the development of cellular models that describe how persistent sodium currents could underlie spontaneous inspiratory bursting in single neurons (Butera et al., 1999). Recent work on the bursting mechanisms of the isolated pre-BötC has highlighted that its small-world connectivity, rather than its intrinsic conductances, underlies the capacity to generate inspiratory bursting activity (Kam et al., 2013a,b). This conceptual model has been incorporated into network models that consist of excitatory neurons containing a subset of spontaneously bursting units connected in a small-world pattern, which is now considered to explain the inspiratory bursting of the isolated pre-BötC (Shao et al., 2006; Ashhad et al., 2023). Beyond the pre-BötC inspiratory activity, several network models have been developed to formalize the long-standing conceptual view of the respiratory network as a central pattern generator (Ogilvie et al., 1992; Rybak et al., 1997; Rybak et al., 2007; Rubin et al., 2009; Gottschalk et al., 1994; Wyman, 1977). These central pattern generator models consist of neurons with mutual inhibitory interactions that sculpt respiratory neuronal activities from several sources of excitatory drive. Importantly, they have shown that reciprocal inhibition can account for a variety of experimental observations including the generation of the three-phase respiratory motor pattern of inspiration, post-inspiration and late-expiration. However, a limitation of previous models is that the respiratory network is not composed of strictly inhibitory or excitatory neurons. For the case of central pattern generator models, this property is highlighted by studies which show that blockade of synaptic inhibition in key inspiratory or expiratory compartments of the respiratory network is not sufficient to ablate the respiratory pattern *in vivo* (Ashhad et al., 2022; Janczewski et al., 2013). Thus, there remains a need for computational models of respiratory rhythm and pattern formation that have greater face validity.

In the present study, we developed a network model of respiratory rhythmogenesis that incorporated excitatory, inhibitory and neuromodulatory connections. Using previously proposed connectivity patterns (Kleinfeld and Sompolinsky, 1988; Kleinfeld and Sompolinsky, 1989), we were able to generate a network model of respiratory rhythm and pattern generation based on the assumed set of respiratory firing patterns which we measured from ensemble recordings of the pre-BötC *in situ*. This model encapsulated our hypothesis that asymmetric neuromodulatory connections can promote the generation of the respiratory rhythm. In testing this core assumption, we found that perturbations of the model connectivity weights were consistent with experimental perturbations of serotonergic or opioidergic neurotransmission. However, the model is not without limitation.

One critical issue is the mapping between slow synaptic effects in the model and neuromodulatory signaling *in situ*. While neuromodulatory transmitters act via metabotropic receptors to slowly modulate membrane potential, they are not the only biophysical process that can produce slow rhythmic fluctuations in membrane potential. For instance, in forebrain networks, ion concentrations modulated by intrinsic membrane pumps and glial activities have been proposed to cause the slow, resting-state fluctuations in membrane potentials (Krishnan et al., 2018). In the respiratory network, subsets of spontaneously bursting pre-BotC neurons express persistent sodium channels that mediate the slow depolarization of their membrane potential (Butera et al., 1999; Rybak et al., 2007). These observations raise the possibility that the slow synapses in the model may not map to neuromodulation. However, in our *in situ* experiments, we explicitly perturbed neuromodulatory signaling with *μ*-OR and 5HT1a agonists and observed responses consistent with corresponding *in silico* simulations. Therefore, it is likely that the slow synaptic weights in the model map to neuromodulatory synapses in the biologic respiratory network.

This raises an additional question of how generalizable these pharmacologic perturbations are: do all neuromodulatory receptor agonists lead to an effect that resembles that of increasing the weights of slow-inhibition or excitation in the model? For the case of increasing slow-inhibition, similar subtle increases in respiratory frequency have been widely observed in pharmacologic, optogenetic or chemogenetic experiments both in reduced slice preparations *in vitro* and in the intact network *in situ* for many neuromodulatory systems (Doi and Ramirez, 2008) including, for example, serotonin (Richter et al., 2003; DePuy et al., 2011; Manzke et al., 2003), acetylcholine (Shao and Feldman, 2005; Shao and Feldman, 2000), norepinephrine (Viemari and Ramirez, 2006; Viemari and Tryba, 2009), dopamine (Fujii et al., 2004), ATP (Sheikhbahaei et al., 2018; Gourine et al., 2010; Gourine et al., 2005) and histamine (Dutschmann et al., 2003). Another related question is whether similar changes in respiratory dynamics would occur using a pharmacologic approach that aimed to block endogenous neuromodulatory transmission rather than the agonist experiments described here which evoke exogenous neuromodulatory transmission. In the case of neuromodulation by acetylcholine, a previous study has demonstrated that blockade of muscarinic receptors increased respiratory frequency in the *in vitro* slice preparation, an effect that was subsequently reversed by bath application of atropine (Shao and Feldman, 2000). However, whether similar effects on respiratory network activity would be observed in the intact network, like that of the *in situ* arterially-perfused brainstem-spinal cord preparation, remains an important question for future investigation.

Other limitations of the model include its lack of spontaneous bursting neurons, the simplified dynamics of Hopfield units and its violation of Dale’s law. While the model does not include spontaneous bursting neurons, spontaneous bursting neurons have been shown to be dispensable in central pattern generator models of the respiratory rhythm (Rubin and Smith, 2019). Second, the Hopfield units of our model are binary and thus cannot generate the spiking or bursting dynamics associated with more detailed neuron models. Further, Hopfield units cannot describe biologic neurons that can fire at vastly different firing rates. However, it was demonstrated that the network connectivity patterns of Hopfield networks can be translated into those for spiking networks to yield networks with similar behavior (Maass and Natschläger, 1997). Finally, the present model also does not follow Dale’s law since the Hopfield units can have both excitatory and inhibitory neurons. Nonetheless, given such significant simplifications compared to the biological system, it is notable that the model was able to explain both the collapse of network activity following fentanyl administration and the frequency increase evoked by 5HT1a-mediated neuromodulation.

### Population coding of respiratory phase transitions

The findings of the present study also extend previous observations of a population code of respiratory phase transitions. In an earlier study, we reported that respiratory LFPs, which reflect the synaptic activities of local populations, peaked specifically at the transitions between the three phases of the respiratory cycle throughout the ponto-medullary respiratory network (Dhingra et al., 2020). In the present study, this feature was observed both in the model in the ensemble activity of the pre-BotC *in situ*. In the model, transitions between states are evoked by slow neuromodulatory transmission that acts to change the ‘energy’ landscape of the network such that the fixed point associated with a given state is destabilized in the direction of the fixed point associated with the next state (Kleinfeld and Sompolinsky, 1989). At the transition, a partial cue of the next state’s memory is established allowing the network to recall the activity pattern of the next state. These transitions involve a brief overlap of the activities of adjacent Hebbian assemblies as the memory of the next fixed point is recalled and stabilized. Interestingly, peaks in the network’s population activity appeared at only three of eight transitions, which corresponded to those between inspiration, post-inspiration and late-expiration. We observed a qualitatively similar pattern of population activity in pre-BötC ensembles *in situ*. Importantly, this property of population activity cannot be accounted for in half-center oscillator CPG models of respiratory pattern generation (Supplementary Figure 3) since transitions in such models occur via an escape or release mechanism in which the population activity at a transition is either balanced or shifts to a new plateau depending on the number of units active before and after the transition (Rubin et al., 2009). Thus, the proposed model of asymmetric neuromodulation better accounts for the population activity of the respiratory network *in situ*.

In addition, we observed, both in the model and in experiments, that the cycle-triggered average of the population activity in the network or in the pre-BötC, respectively, resembled the bi-phasic discharge of the vagal motor pattern, which regulates upper-airway patency. This observation is consistent with the recent characterization of a role for the pre-BötC in regulating, not just inspiratory discharge in the inspiratory motor nerves, but also in the inspiratory and post-inspiratory activity in the vagus (Dhingra et al., 2024). In the model, this bi-phasic pattern of population activity reflects the distribution of firing patterns the network was trained to generate. We derived this distribution directly from the clustering of firing patterns present in ensemble recordings *in situ*. Consistent with previous observations in the intact brainstem, the distribution of pre-BötC firing patterns included neurons with bursting activity in the inspiratory, post-inspiratory and late-expiratory phases as well as neurons with tonic or tonic respiratory modulated activities (Connelly et al., 1992; Schwarzacher et al., 1995; Sun et al., 1998). The latter classes of respiratory neurons have been previously implicated in respiratory phase switching and the reflex and behavioral control of the respiratory pattern (Cohen et al., 1993; Cohen, 1973; Segers et al., 2015; Orem, 1989). In contrast, in our model, these patterns of activity are merely a consequence of the overall network connectivity, with each population’s slow synapses playing significant roles in determining respiratory phase switching. Consistent with this experimental finding, we observed a stronger cross-correlation between pre-BötC ensemble activity and the vagal motor pattern than the activity of any individual pre-BötC neuron suggesting that the population, rather than individual bursting neurons, is responsible for encoding the respiratory motor pattern in network activity. Together, these experimental data are consistent with the temporal structure of the model network’s population activity.

### Implications for opioid-induced respiratory depression (OIRD)

OIRD remains a significant health problem in the United States (Ramirez et al., 2021; Dahan et al., 2024). Recently, the risk posed by illicit synthetic μ-OR agonists has been further exacerbated by the presence of adulterants like xylazine that act on α2 adrenergic receptors and nitazenes which are μ-OR agonists that may not be fully counteracted by the μ-OR antagonist naloxone (Dahan et al., 2024). Thus, the incidence of OIRD due to synthetic opioids and combinations of opioid and non-opioid substances has motivated the need to discover new therapeutics to counteract OIRD. Our computational model and experimental results suggest that neuromodulatory connectivity within the respiratory network is organized asymmetrically to promote rhythmogenesis. We propose that the pattern of neuromodulation should be considered for the rational design of therapies to treat respiratory disorders like OIRD. Further, our results suggest that identification of alternative neuromodulatory targets to prevent or reverse OIRD will require the consideration of the pattern of neuromodulator receptor expression, its overlap with that of μ-OR expression and the firing pattern of the target respiratory neurons.

One strategy to assess the pattern of neuromodulation would be to monitor respiratory network activity using MEAs in the *in situ* preparation, but with an experimental design to assess the dose response of the drug with respect to respiratory network activity. The dose response experimental strategy would enable one to identify experimentally which neuron types are impacted by fentanyl and to identify the relationship between the units that remain active during persistent apnea with their firing patterns during eupneic breathing. In contrast, in the present study, we induced fentanyl-evoked persistent apnea with a single dose. While this perturbation was qualitatively similar to simulations of the model after increasing slow excitation, it also provides further experimental evidence that opioids do not simply inhibit all pre-BotC neurons to suppress breathing, but instead act through a network mechanism that, according to the model, may involve a net increase in slow excitation across the network.

Neuromodulatory signaling pathways have long been therapeutic targets for respiratory disorders. A remarkable example of this strategy occurred in the case of a patient who experienced severe apneustic respiratory disturbances after surgical resection of a tumor at the ponto-medullary junction (Wilken et al., 1997). In this case study, the apneustic respiratory motor pattern was corrected without side-effects using buspirone, a 5HT1aR agonist. The rationale behind the therapy arose from the perspective that neuromodulators act to influence intracellular second messenger cascades and that counteracting the influence of one pathway could be achieved by activating alternative second messenger systems with the right neuromodulatory agonist (Richter et al., 1997). However, the hypothesis regarding downstream effects on membrane excitability came from intracellular recordings of very few respiratory neurons before and after drug applications. This limited evidence also led to other cases in which neuromodulatory therapies were met with limited success. For instance, 5HT4aRs were identified as a therapeutic to better manage OIRD without the loss of analgesia that accompanies OIRD reversal with naloxone (Manzke et al., 2003). However, later clinical trials showed that the 5HT4R-agonist mosapride was ineffective in recovering from morphine-induced OIRD in humans (Lötsch et al., 2005). In the case of the irregular respiratory rhythms present in patients with Rett syndrome, pre-clinical studies in MeCP2-deficient mice developed strong evidence that drugs targeting serotonergic and dopaminergic receptors were effective to correct respiratory disturbances (Abdala et al., 2013; Abdala et al., 2010). However, clinical trials in Rett patients treated with saritozan, a 5HT1aR- and D2R-agonist, were unsuccessful (Evaluation of the Efficacy, Safety, and Tolerability of Sarizotan in Rett Syndrome with Respiratory Symptoms (STARS), 2021).

Despite the simplicity of this network model, increasing the weights of slow-excitatory neuromodulatory connections were consistent with the pattern of network activity that was experimentally observed following fentanyl-induced OIRD, specifically a reduction in the number of active neurons that spared populations with either tonic or short bursting activities. Importantly, we note that pre-BötC ensemble activity and its collapse post-fentanyl exposure was representative of the network as a whole is well supported by a recent study that observed similar patterns of neuronal activity (tonic and short-bursting populations) in the dorsolateral pons (Saunders et al., 2022). Direct comparison of the findings of the present study with those of another recent study using multi-electrode arrays to monitor medullary respiratory network activity during ORID (Bush and Ramirez, 2024) is difficult because that study used a lower dose of morphine that was not sufficient to evoke the fully collapsed state of network activity that is present during opioid-induced persistent apnea. Nonetheless, their representative raster plots of respiratory network activity show evidence of partially collapsed states of network activity that may be consistent with our model when we only moderately increase slow-excitation (Supplementary Figures 8D,E). We note that unlike OIRD *in vivo*, a mild increase in slow inhibition did not appear to change the overall frequency of the model network’s periodic activities. However, we observed that mild increases in slow excitation in the model evoked an inhibition of subsets of respiratory neurons which was consistent with reported effects of OIRD *in vivo* (Haji et al., 2003; Takeda et al., 2001).

That a relatively simple model of respiratory pattern generation could explain the effects of neuromodulation highlights the need to consider the pattern of neuromodulation across the network for the rational design of neuromodulatory therapies. In other words, one should address the question of whether a proposed neuromodulatory therapeutic targets the opposing asymmetric respiratory neuronal populations to promote respiratory pattern formation? Nonetheless, these simulations and experiments support previous suggestions to develop combinatorial neuromodulatory therapies, particularly to protect against opioid-induced respiratory depression (Richter et al., 2003; Manzke et al., 2003; Dutschmann et al., 2009).

The need to consider the network mechanism of respiratory neuromodulation is further highlighted by the fact that both neuromodulatory agonists used in the present study are coupled to G_i/o_-dependent signaling cascades (Richter et al., 2003; Reeves et al., 2022). In the case of the 5-HT1aRs, our findings were consistent with a predominant effect of slow-inhibition in the network. In the case of μ-ORs, our results which suggest a net effect of opioids to increase slow-excitation may appear counter-intuitive to the commonly held notion that activation of μ-ORs evokes inhibition of membrane excitability. Importantly, the action of a particular neuromodulatory receptor agonist on one cell-type does not necessarily generalize to its effect on any neuron. Instead, the effect of activating neuromodulatory receptors depends on the targets of the corresponding intracellular signaling cascades which vary across neuronal cell types. In the case of μ-ORs, it is well known that neurons can show either excitatory or inhibitory effects depending on the cell-type (Reeves et al., 2022). One simple explanation of our observations is that μ-ORs may have a greater effect on inhibitory neurons such that the net effect of opioids at the level of the respiratory network is that of a slow-disinhibition. Alternatively, it has also been shown that μ-ORs can directly excite their target neurons via the coupling of their G_βγ_-subunits to PLC-dependent signaling cascades that increase intracellular calcium levels (Charles and Hales, 2004). In either case, our computational and experimental observations supporting an asymmetric pattern of neuromodulation in the network further highlight the need to consider the intracellular effects of a neuromodulatory pathway across the whole network, rather than in small subsets of respiratory neurons.

## Data Availability

The raw data supporting the conclusions of this article will be made available by the authors without undue reservation.
