# Supplementary material for: Asymmetric neuromodulation in the respiratory network contributes to rhythm and pattern generation
Source: Front Neural Circuits. 2025 Jul 8;19:1532401. doi: 10.3389/fncir.2025.1532401 (PMC12279826; doi:10.3389/fncir.2025.1532401)
Supplement: Supplementary file 1 [file Data_Sheet_1.pdf]

Supplementary Methods

Registration of neuronal locations in the Waxholm atlas coordinate space

In a subset of experiments, both to confirm the localization of these neuronal types to the pre-BötC and to assess their spatial distribution within the pre-BötC, we developed an approach to determine the rigid transformation that would register the coordinate space of the MEA micro-positioner to the Waxholm 7T MRI atlas of the Sprague-Dawley rat brain (36). To do so, we first measured the coordinates of 5 brainstem surface landmarks of the floor of the 4th ventricle that are readily observable in both the *in situ* preparation and in the Waxholm atlas (Suppl. Fig. 1). Using these correspondence points, we calculated the rigid transformation that would register these two coordinate spaces using the analytic method proposed by (84)(Suppl. Fig. 1b). After registering the MEA recording sites to the Waxholm MRI atlas of the Sprague-Dawley rat brain, we estimated the 'centre-of-mass' of each neuron's spike template to determine the location of each neuron relative to the MEA. Then, we applied the rigid transformation to these MEA unit location coordinates to determine the anatomic location of each neuron within the Waxholm atlas. This method enabled an estimation of the position of the MEA electrode sites within the Waxholm atlas and confirmed that the recorded ensembles were localized to the pre-BötC (Fig. 1a & f).

Respiratory CPG model

To illustrate the population activity associated with half-centre oscillator models of respiratory pattern generation, we simulated the respiratory CPG model of (49). The model consists of four neurons representing pre-I, early-I, post-I and aug-E populations. These neuronal populations were connected via reciprocal inhibition and received common excitatory drive from three sources. The membrane potential of the pre-I neuron V_1 is intrinsically bursting due to its persistent sodium current and was defined by the following equation:

$$\mathrm{C}\,\frac{dV_1}{dt} = -\mathrm{I_{NaP}} - \mathrm{I_K} - \mathrm{I_{L_1}} - \mathrm{I_{SynE_1}} - \mathrm{I_{SynI_1}}$$

where I_{NaP} is the persistent sodium current and I_K is the delayed rectifier potassium current.

The membrane potential of the other neurons ($i \in 2{,}3{,}4$) was

$$C\,\frac{dV_i}{dt} = -I_{AD_i} - I_{L_i} - I_{SynE_i} - I_{SynI_i}$$

where I_{AD_i} is an outward potassium current that mediates adaptation behaviour. In both of the above equations, C is the membrane capacitance, I_{L_i} are the leak currents, I_{SynE_i} and I_{SynI_i} are the excitatory and inhibitory synaptic currents.

The membrane currents are defined by the following equations

$$I_{NaP} = \overline{g}_{NaP} m_{NaP} h_{NaP} (V_1 - E_{Na})$$

$$I_K = \overline{g}_K m_K^4 (V_1 - E_K)$$

$$I_{AD_i} = \overline{g}_{AD} m_{AD_i} (V_i - E_K)$$

$$I_{L_i} = \overline{g}_L (V_i - E_L)$$

$$I_{SynE_i} = \overline{g}_{SynE} \left(V_i - E_{SynE}\right) \sum_{k=1}^{3} c_{ki}\, d_k \quad \forall\ i \neq 2$$

$$I_{SynE_2} = \overline{g}_{SynE}(V_2 - E_{SynE})\left[a_{12}f_1(V_1) + \sum_{k=1}^{3} c_{ki}\, d_k\right]$$

$$I_{SynI} = \overline{g}_{SynI}(V_i - E_{SynI})\sum_{j=2}^{4} b_{ji}\, f_j(V_j) \quad \forall\ j \neq i$$

where $\overline{g}_{NaP}$, $\overline{g}_K$, $\overline{g}_{AD}$, $\overline{g}_L$, $\overline{g}_{SynE}$ and $\overline{g}_{SynI}$ are the maximal conductances of the corresponding currents, E_{Na}, E_K, E_{AD}, E_L, E_{SynE} and E_{SynI} are the corresponding reversal potentials, a_{12} is the synaptic weight from the pre-I to early-I neuron, b_{ji} is the inhibitory synaptic weight from neuron j to neuron i, and c_{ki} is the weight of the synaptic drive from drive d_k ($k \in 1,2,3$) to neuron i.

The nonlinear function $f_i(V_i)$ defines the spiking activity of each neuron

$$\mathrm{f_i(V_i)} = \frac{1}{1 + \mathrm{e}^{\frac{-(\mathrm{V_i} - \mathrm{V_{1/2}})}{\mathrm{k_{V_i}}}}} \quad \forall\ \mathrm{i} \in 1,2,3,4$$

where $V_{1/2}$ is the half-activity voltage and k_{V_i} defines the slope of output function of each neuron.

The slow inactivation of the persistent sodium current is

$$\tau_{h_{NaP}}(V_1)\frac{d}{dt}h_{NaP} = h_{\infty NaP}(V_1) - h_{NaP}$$

The slow adaptation of the other three neurons are

$$\tau_{AD_i}\frac{d}{dt}m_{AD_i} = k_{AD_i}f_i(V_i) - m_{AD_i}$$

where τ_{AD_i} is a fixed time constant and k_{AD_i} is the maximal adaptation.

The voltage-dependent activation and inactivation variables and time constant for the persistent sodium and rectifying potassium channels of neuron 1 are

$$m_{NaP} = \frac{1}{1 + e^{-(V_1+40)/6}}$$

$$\mathrm{h_{\infty NaP}} = \frac{1}{1 + \mathrm{e}^{(\mathrm{V_1}+48)/6}}$$

$$\tau_{h_{NaP}} = \frac{\tau_{h_{NaP}max}}{cosh(V_1 + 48)/12}$$

$$m_K = \frac{1}{1 + e^{-(V_1+29)/4}}$$

Model parameters are defined in Supplementary Table 1. The model was numerically integrated using the fourth-order Runge-Kutta method with a timestep size of 1 ms. After simulation, we computed the population firing rate by summing the firing rates of the neurons, $f_i(V_i)$, and scaling by the network size, $n = 4$.

Supplementary Table 1: Respiratory CPG model parameters.

**Parameter (unit)**	**Values**
Membrane capacitance (pF)	$C = 20$

Maximal conductances (nS)	$\overline{g}_{NaP} = 5.0$, $\overline{g}_{K} = 5.0$, $\overline{g}_{AD} = 10.0$, $\overline{g}_{L} = 2.8$, $\overline{g}_{SynE} = 10.0$ and $\overline{g}_{SynI} = 60.0$
Reversal potentials (mV)	$E_{Na} = 50$, $E_K = -85$, $E_{AD} = -60$, $E_L = -60$, $E_{SynE} = 0$ and $E_{SynI} = -75$
Synaptic weights	$a_{12} = 0.4$, $b_{21} = 0$, $b_{23} = 0.25$, $b_{24} = 0.35$, $b_{31} = 0.3$, $b_{32} = 0.05$, $b_{34} = 0.35$, $b_{41} = 0.2$, $b_{42} = 0.35$, $b_{43} = 0.1$, $c_{11} = 0.115$, $c_{12} = 0.3$, $c_{13} = 0.63$, $c_{14} = 0.33$, $c_{21} = 0.07$, $c_{22} = 0.3$, $c_{23} = 0$, $c_{24} = 0.4$, $c_{31} = 0.025$, $c_{32} = c_{33} = c_{34} = 0$
Parameters of spiking activity functions (mV)	$V_{1/2} = -30$, $k_{V_1} = 8$, $k_{V_2} = k_{V_3} = k_{V_4} = 4$
Time constants (ms)	$\tau_{h_{NaP}max} = 6000$, $\tau_{AD_2} = \tau_{AD_4} = 2000$, $\tau_{AD_3} = 1000$
Adaptation parameters	$k_{AD_2} = k_{AD_4} = 0.9$, $k_{AD_3} = 1.3$

Supplementary Figures

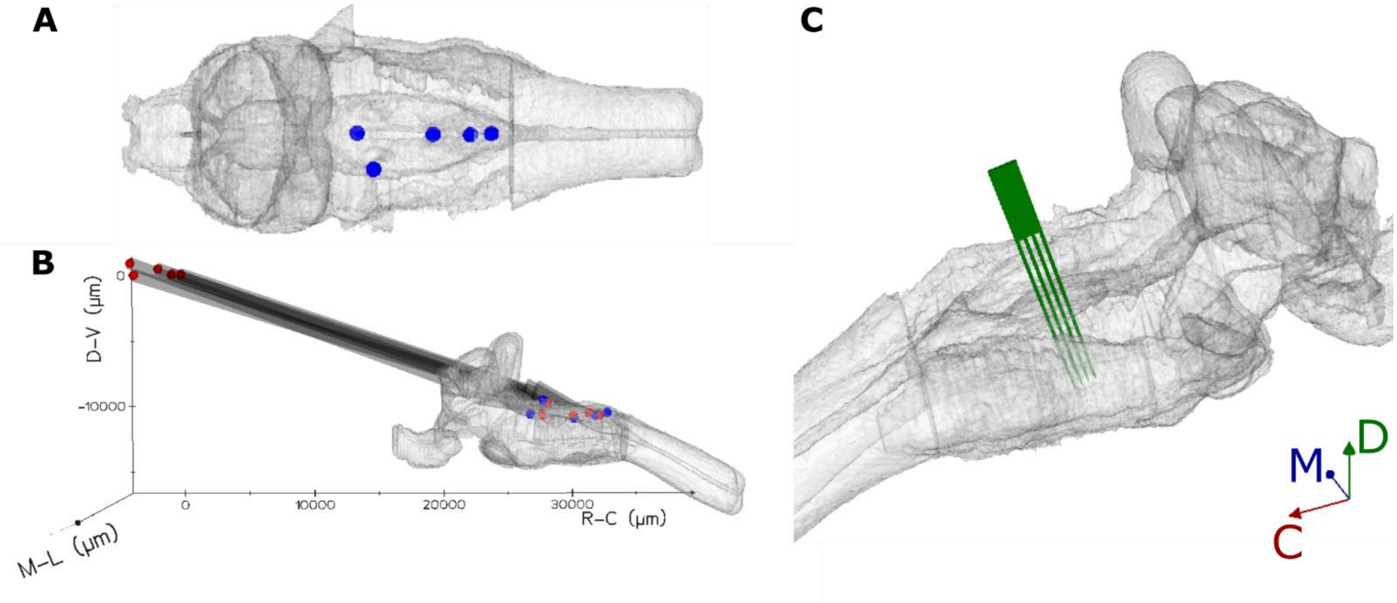

Supplementary Figure 1: Registration of MEA coordinate space to Waxholm atlas of Sprague-Dawley rat brain.

A Locations of brainstem surface landmarks in the Waxholm atlas of the Sprague-Dawley rat brain.

B Registration of the observed surface landmark coordinates (red) to those of the Waxholm atlas (blue).

C Reconstructed MEA positioning in a representative experiment.

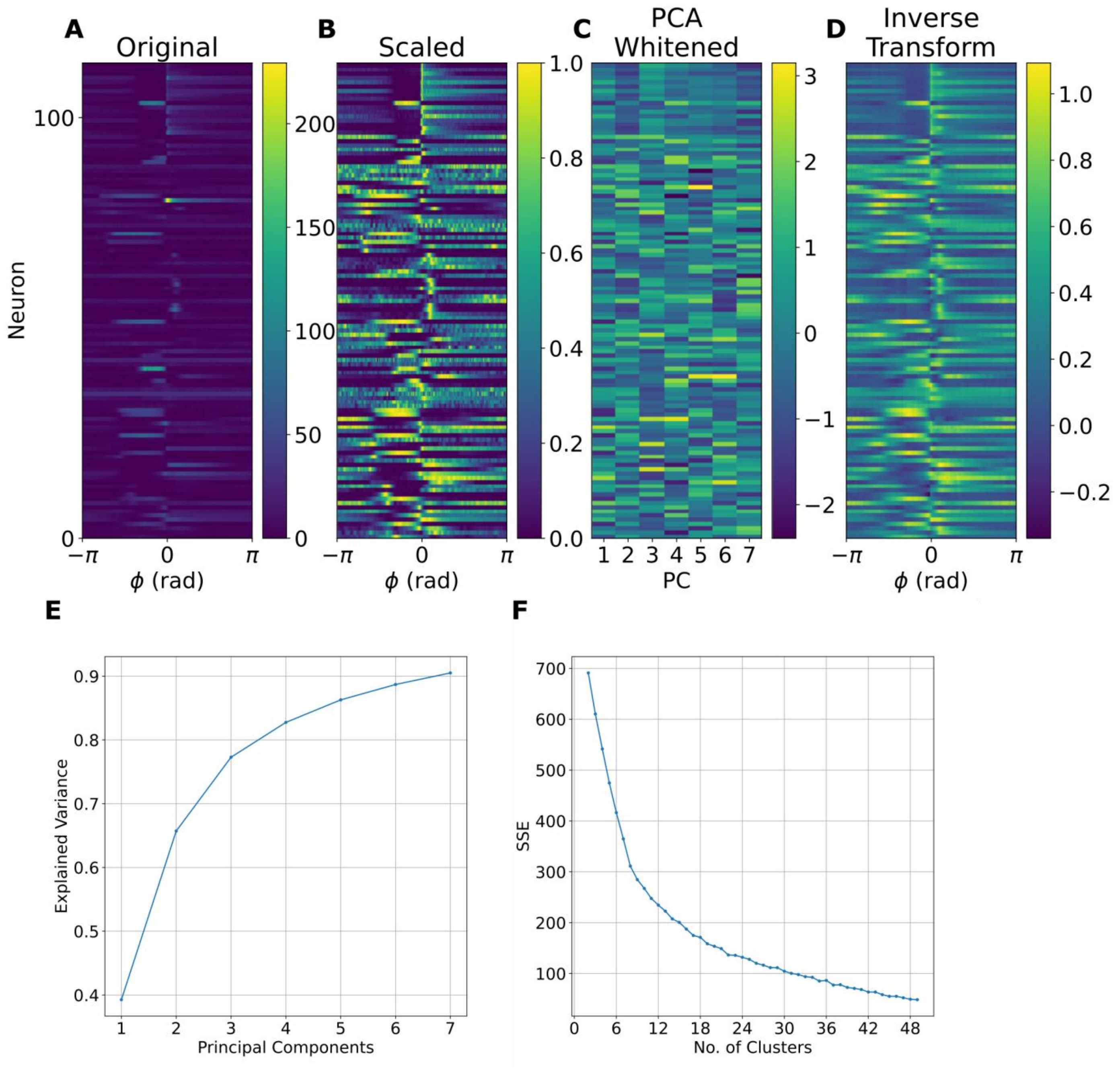

Supplementary Figure 2: Pre-processing of cycle-triggered histograms for k-means clustering.

A Cycle-triggered histograms (CTHs) for all pre-BötC neurons in Hz.

B Scaled CTHs for all pre-BötC neurons in a.u.

C The dimensionality of the dataset was reduced with a PCA keeping the top 7 components which accounted for ~90% of the original variance.

D Inverse transform of the dimensionality reduced dataset shows that no meaningful information about the cycle-triggered firing rate patterns was lost by discarding the bottom principal components.

E The cumulative explained variance is plotted for the first 7 principal components. 7 principal components were required to explain >90% of the variance in the original dataset. Explained variance: cumulative explained variance.

F The within cluster sum of squared errors is plotted for the k-means clustering of the dimensionality reduced dataset for all $k \in [1 - 50]$. We observed the 'elbow' of this plot at $k = 14$. The resultant k-means clustering for this value of k was also consistent with previous classifications of pre-BotC neuronal types (see Fig. 1E). SSE: Within-cluster sum of squared errors; No. of clusters: k.

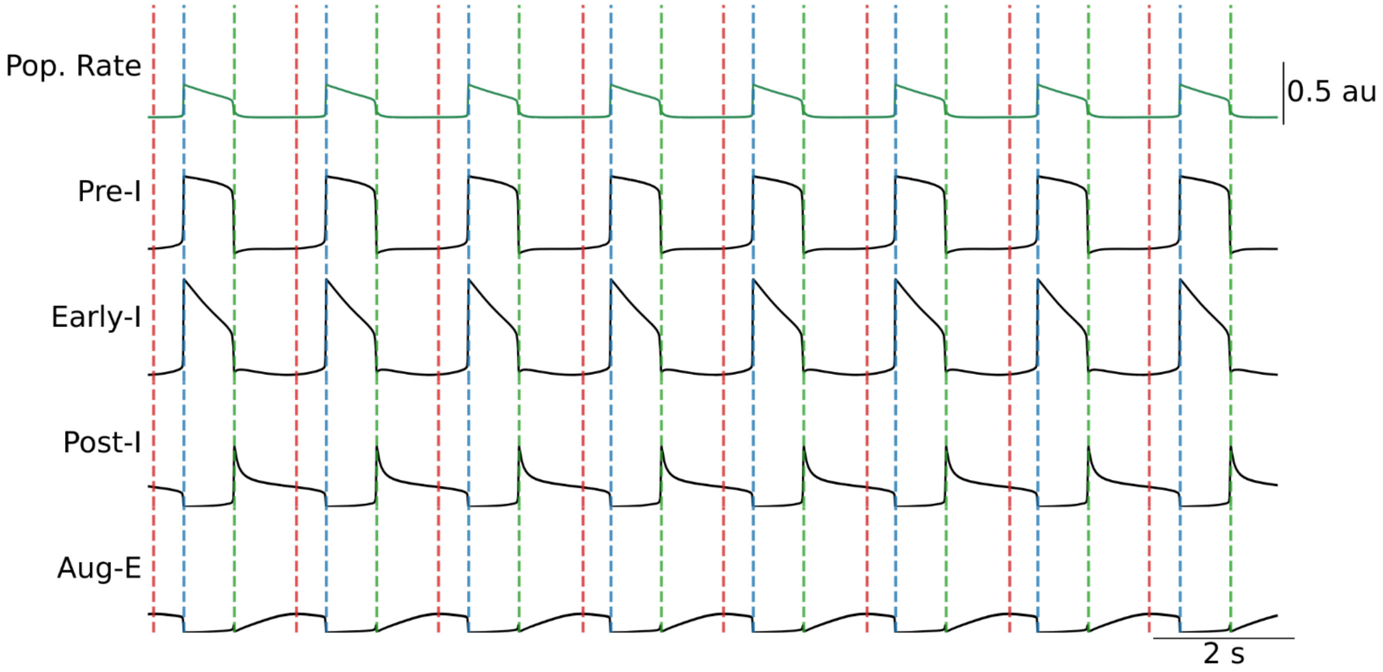

Supplementary Figure 3: A representative CPG model of respiratory pattern generation does not encode respiratory phase transitions in population activity.

To illustrate the lack of population coding in respiratory CPG models, we simulated the respiratory CPG model of (49) as described and measured its population firing rate (green, Pop. Rate). As expected, because phase transitions in CPG models involve an escape or release mechanism, the population firing rate at transitions between respiratory phases was either balanced (PI-E2 transition, red dashed lines) or involved a transition to a new plateau (E2-I and I-PI transitions, blue- and green-dashed lines, respectively).

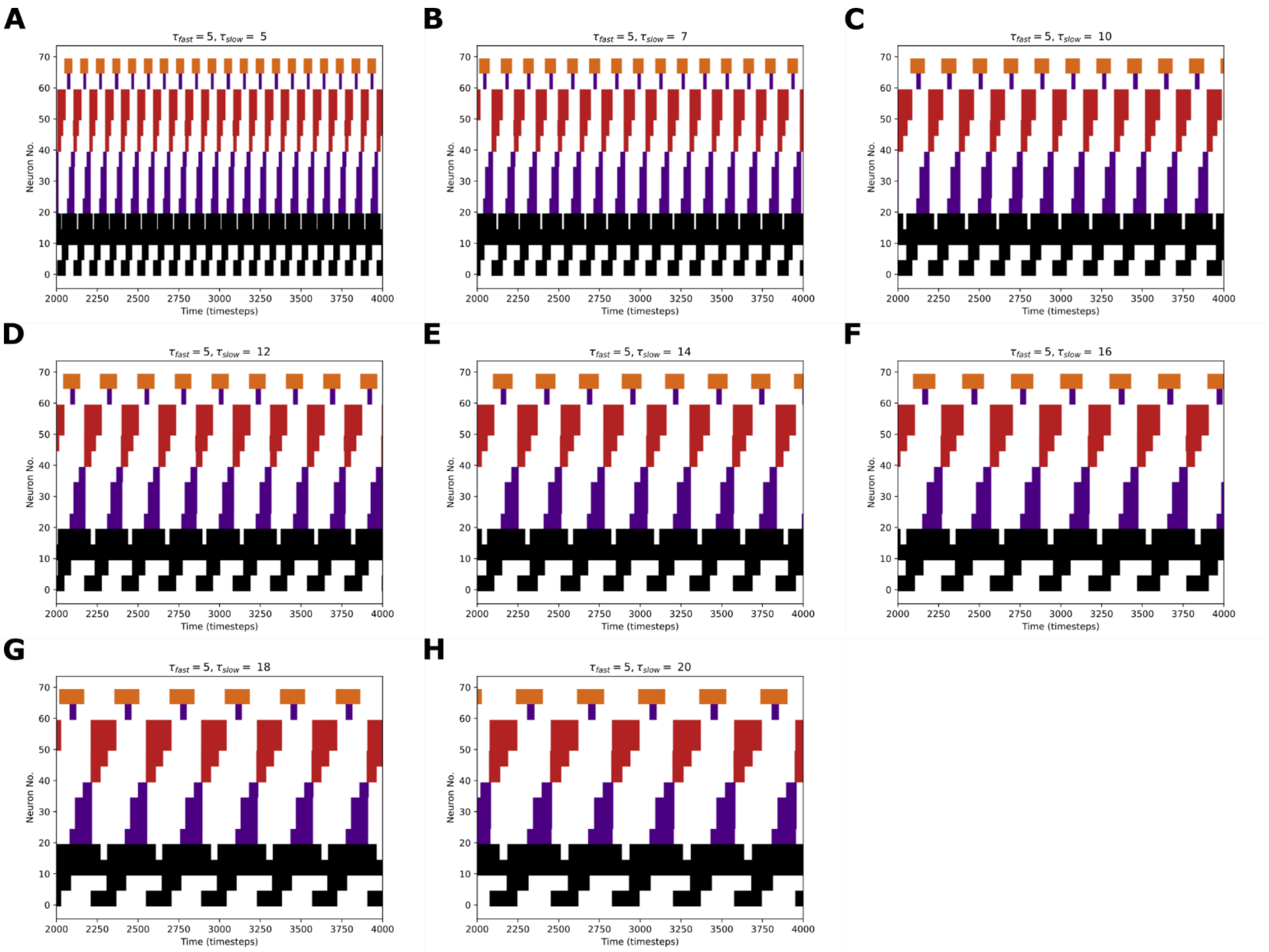

Supplementary Figure 4: Increasing τ_{slow} while keeping τ_{fast} fixed increases duration spent in each sequential state. As described by Kleinfeld & Sompolinsky (29), the ratio of the fast and slow time constants determines the time spent in each state before a transition occurs.

A-H We increased τ_{slow} while keeping τ_{fast} and all other network parameters fixed to illustrate this basic property of Hopfield networks with fast and slow synapses. The parameter values associated with each simulation are shown above each plot.

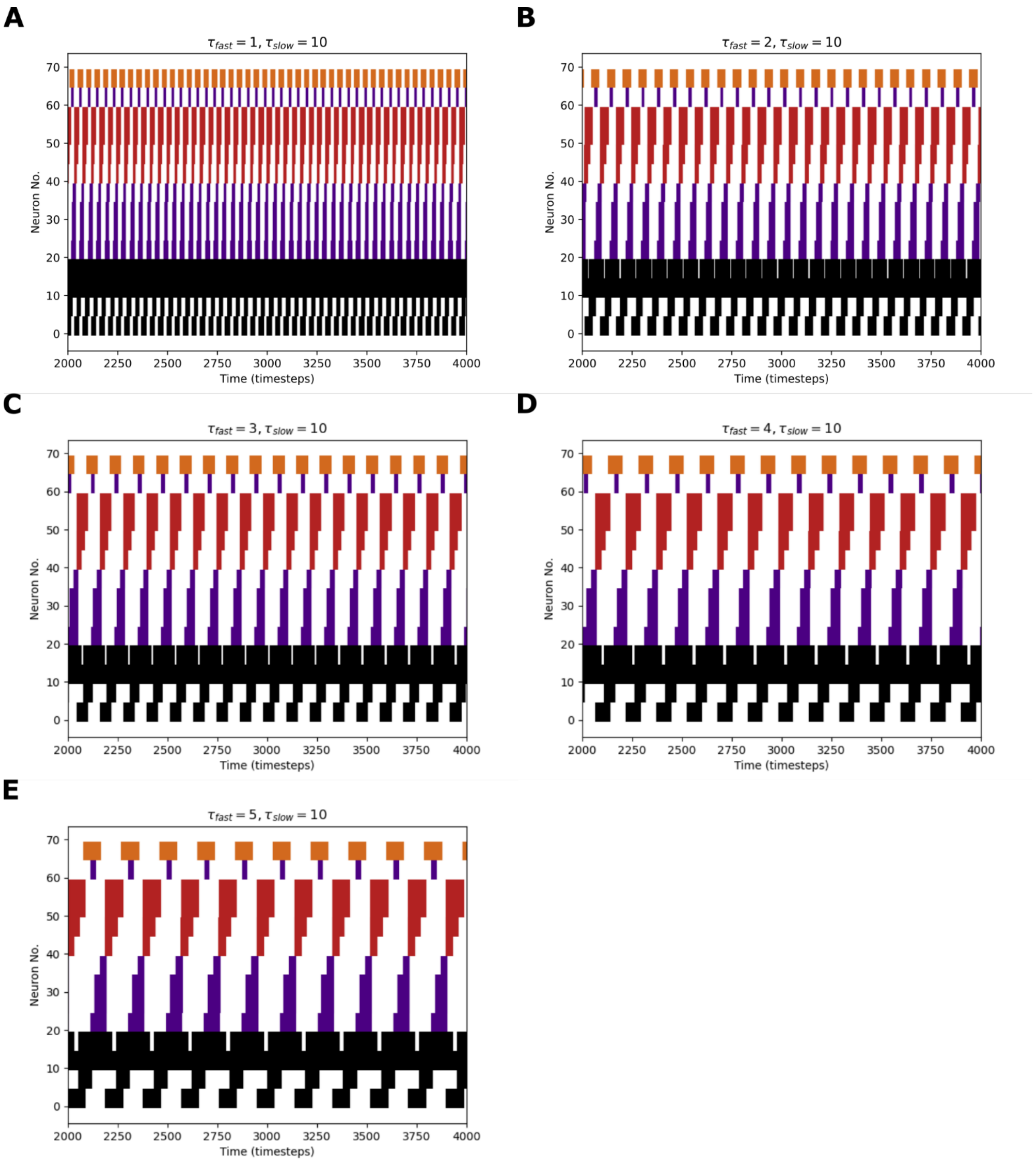

Supplementary Figure 5: Increasing τ_{fast} while keeping τ_{slow} fixed increases duration spent in each sequential state. As described by Kleinfeld & Sompolinsky (29), the ratio of the fast and slow time constants determines the time spent in each state before a transition occurs.

A-E We increased τ_{fast} while keeping τ_{slow} and all other network parameters fixed to illustrate this basic property of Hopfield networks with fast and slow synapses. The parameter values associated with each simulation are shown above each plot.

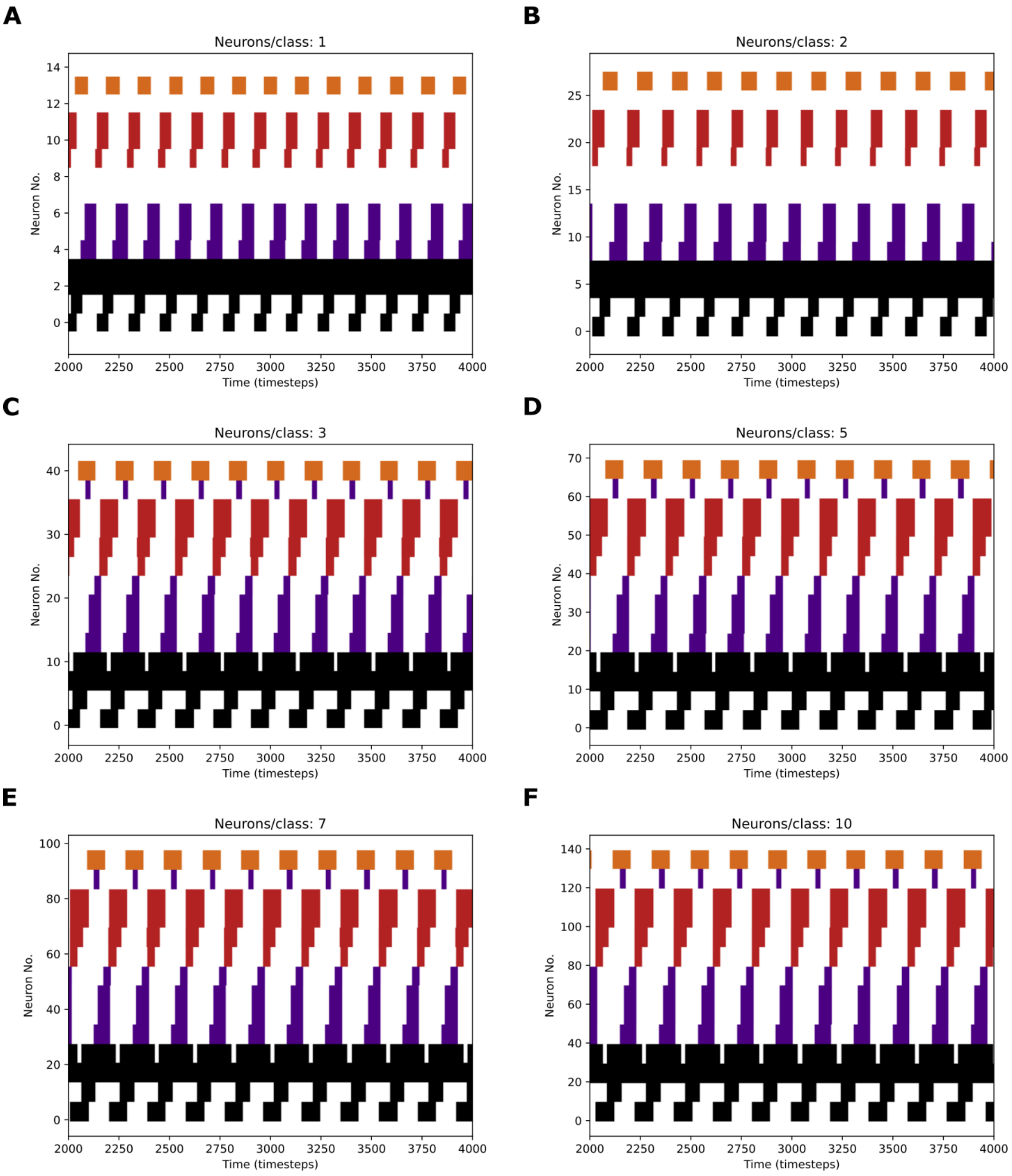

Supplementary Figure 6: Effect of changing the number of neurons/class on network activity.

A-F The number of units in a Hopfield network determines its memory capacity. Here, we illustrate that at least 42 Hopfield units were required to faithfully encode the states that correspond to the periodic sequential activities present in the respiratory network. We increased the number of neurons/class from 1 to 10 neurons/class. Below 3 neurons/class (**A & B**), the network could not faithfully encode the respiratory

network's sequential firing patterns. With at least 3 or more Hopfield units/class (**C-F**), the network was able to encode the sequential firing patterns specified.

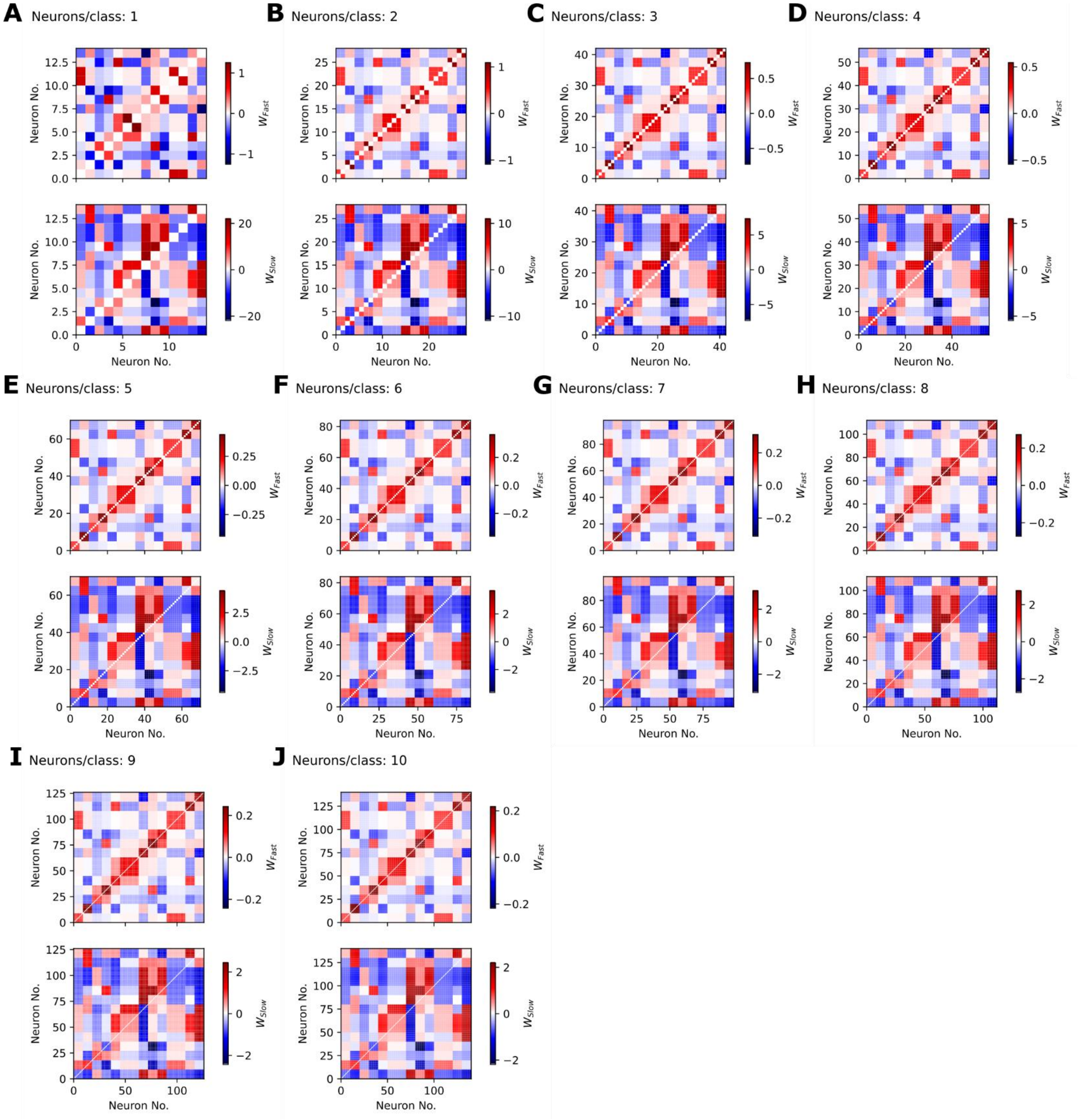

Supplementary Figure 7: Effect of changing number of neurons/class on network connectivity.

A-J For each number of neurons/class, we determined the network's fast (w_{Fast}) and slow (w_{Slow}) connectivity as described in Methods (Eqns. 7-10). As expected from Eqns. 9 & 10, changing the number of neurons/class simply changes the scaling of the synaptic weights (via the term J_0/N, where N is the number of neurons in the network) without changing the pattern of the network's fast or slow connectivity.

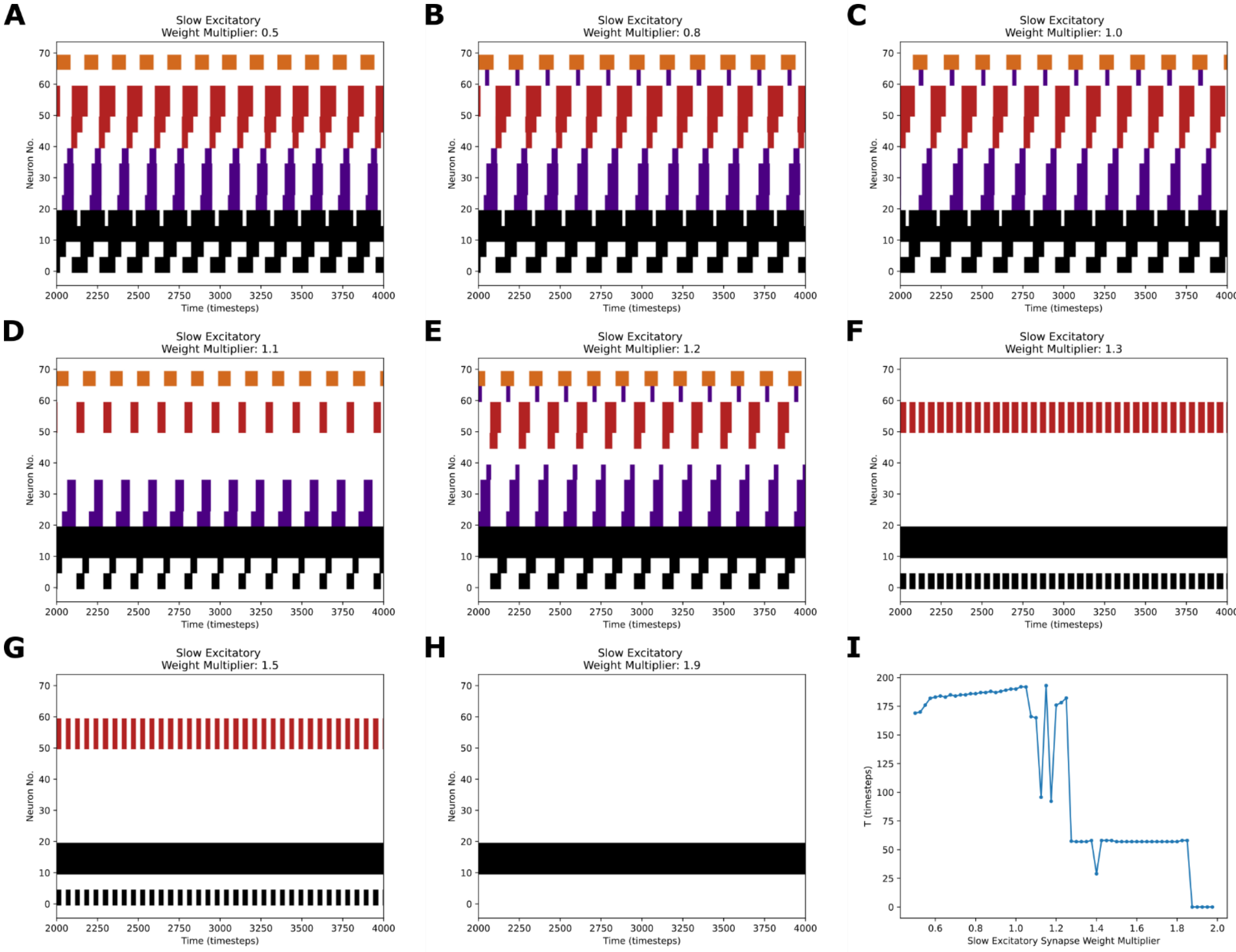

Supplementary Figure 8: Effect of changing slow-, excitatory-synaptic weight multiplier on network dynamics.

A-C Below the baseline values, uniformly decreasing the slow excitatory synaptic weights (<1) does not change the pattern or period of the network oscillation.

D-E For smaller increases in slow excitatory synaptic weights (1.1 – 1.3), some populations become inhibited while the period of the respiratory is similar to baseline (see **I**).

F-G The slow excitatory synaptic weight multipliers between 1.3 and 1.9 have the collapsed pattern of network activity as described in Results & Figures 3 & 5.

H For slow excitatory synaptic weight multipliers greater than 1.9, there is another level of network collapse that only spares tonically active units. In this study, we focused on the first level of network collapse (**F-G**) because physiologically, it is at this first transition, when the respiratory motor pattern transitions from opioid-induced respiratory depression to opioid-induced persistent apnea, the event that leads to significant morbidity after exposure to ultra potent synthetic opioids.

I To quantify the changes in the network rhythm as slow-excitation is increased, we measured the period in the oscillation of unit 3 of the Tonic A sub-population.

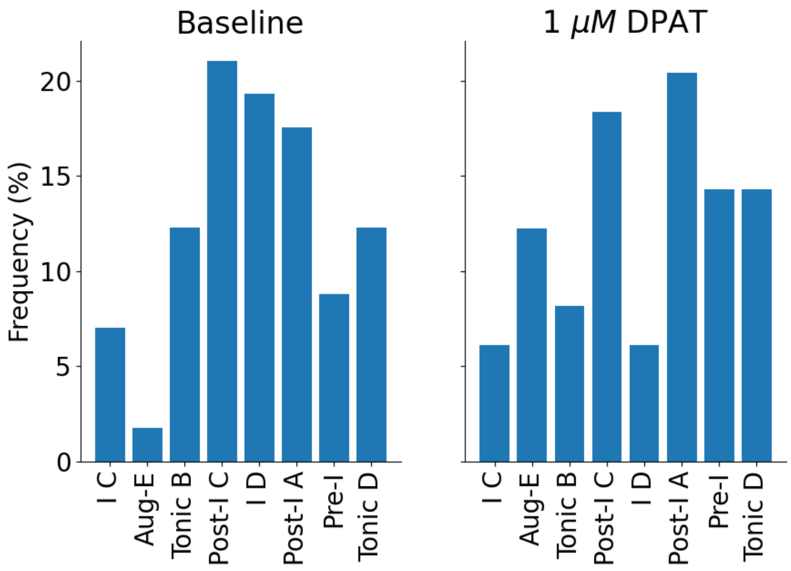

Supplementary Figure 9: Distributions of respiratory neuronal firing patterns observed before and after systemic administration of the 5HT1AR agonist 8-OH DPAT.

Because there was an apparent reconfiguration of pre-BötC neuronal activities evoked by 8-OH DPAT administration, evaluation of the model predictions of increasing slow inhibition in the network required addressing the question of whether the distribution of respiratory neuronal firing patterns was altered by 8-OH DPAT administration. To do so, we used a Fisher's exact test to compare these distributions. As described in Results, with a p-value of 0.232, we rejected the null hypothesis that the distributions were significantly different and concluded that 8-OH DPAT did not change the distribution of respiratory neuronal firing patterns despite the apparent reconfiguration of pre-BötC ensemble activities.

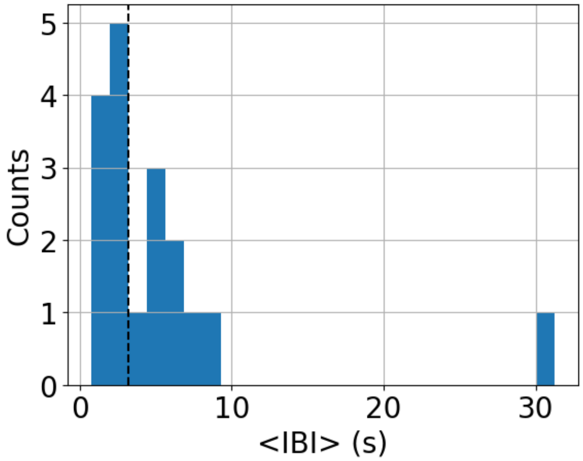

Supplemental Figure 10: Distribution of the inter-burst intervals of bursting neurons whose activity persists following fentanyl-evoked persistent apnea.

The distribution of inter-burst intervals following fentanyl-evoked persistent apnea appeared bi-modal. Thus, to assess the qualitative similarities between these sub-populations of bursting neurons and those of the model, we sub-divided these groups based on the median inter-burst interval, indicated by a black dashed line, for further analysis.

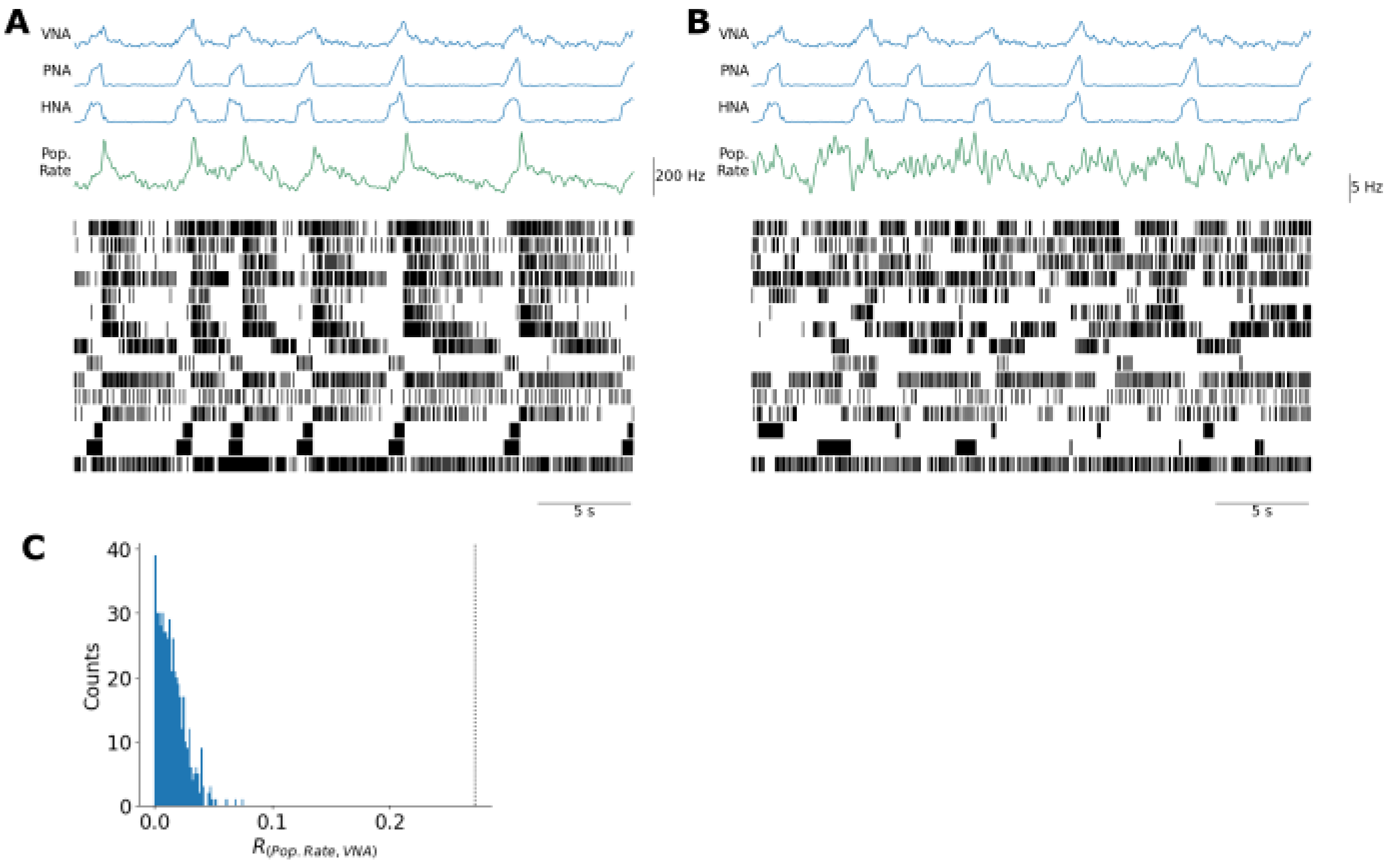

Supplementary Figure 11: Bootstrapping population firing rate measures.

A A representative example of a pre-BötC ensemble, its population firing rate (Pop. Rate, *green trace*) and the respiratory motor pattern on vagal (VNA), phrenic (PNA) and hypoglossal (HNA) nerves.

B A representative example of a shuffled ensemble. To generate a surrogate dataset, we shuffled the inter-spike intervals of each unit in the ensemble, and computed the population firing rate of the shuffled ensemble (Pop. Rate, *green trace*). This shuffling was repeated 500 times.

C The distribution of the cross-correlation of the shuffled ensemble's population firing rate with VNA (*blue*). The cross-correlation of the original ensemble's population firing rate with VNA is indicated by the black dotted line.
